# *Helicobacter pylori-*induced aberrant demethylation and expression of GNB4 promotes gastric carcinogenesis via the Hippo–YAP1 pathway

**DOI:** 10.1186/s12916-023-02842-6

**Published:** 2023-04-05

**Authors:** Duanrui Liu, Yunyun Liu, Wenshuai Zhu, Yi Lu, Jingyu Zhu, Xiaoli Ma, Yuanxin Xing, Mingjie Yuan, Bin Ning, Yunshan Wang, Yanfei Jia

**Affiliations:** 1 Department of Clinical Laboratory, Shandong Provincial Hospital Affiliated to Shandong First Medical University, Jinan, 250021 People’s Republic of China; 2 Research Center of Basic Medicine, Jinan Central Hospital, Shandong University, Jinan, 250013 People’s Republic of China; 3 Research Center of Basic Medicine, Jinan Central Hospital, Shandong First Medical University, Jinan, 250013 People’s Republic of China; 4 Department of Gastroenterology, Jinan Central Hospital, Shandong First Medical University, Jinan, 250013 People’s Republic of China; 5 Central Hospital Affiliated to Shandong First Medical University, Shandong First Medical University, Jinan, 250013 People’s Republic of China

**Keywords:** Gastric cancer, *Helicobacter pylori*, TET1, DNA demethylation, Hippo–YAP1 pathway

## Abstract

**Background:**

*Helicobacter* *pylori* (*H. pylori*) infection causes aberrant DNA methylation and contributes to the risk of gastric cancer (GC). Guanine nucleotide-binding protein subunit beta-4 (GNB4) is involved in various tumorigenic processes. We found an aberrant methylation level of GNB4 in *H. pylori*-induced GC in our previous bioinformatic analysis; however, its expression and underlying molecular mechanisms are poorly understood.

**Methods:**

The expression, underlying signaling pathways, and clinical significance of GNB4 were analyzed in a local cohort of 107 patients with GC and several public databases. *H. pylori* infection was induced in in vitro and in vivo models. Methylation-specific PCR, pyrosequencing, and mass spectrometry analysis were used to detect changes in methylation levels. GNB4, TET1, and YAP1 were overexpressed or knocked down in GC cell lines. We performed gain- and loss-of-function experiments, including CCK-8, EdU, colony formation, transwell migration, and invasion assays. Nude mice were injected with genetically manipulated GC cells, and the growth of xenograft tumors and metastases was measured. Real-time quantitative PCR, western blotting, immunofluorescence, immunohistochemistry, chromatin immunoprecipitation, and co-immunoprecipitation experiments were performed to elucidate the underlying molecular mechanisms.

**Results:**

GNB4 expression was significantly upregulated in GC and correlated with aggressive clinical characteristics and poor prognosis. Increased levels of GNB4 were associated with shorter survival times. Infection with *H. pylori* strains 26695 and SS1 induced GNB4 mRNA and protein expression in GC cell lines and mice. Additionally, silencing of GNB4 blocked the pro-proliferative, metastatic, and invasive ability of *H. pylori* in GC cells. *H. pylori* infection remarkably decreased the methylation level of the GNB4 promoter region, particularly at the CpG#5 site (chr3:179451746–179451745). *H. pylori* infection upregulated TET1 expression via activation of the NF-κB. TET binds to the GNB4 promoter region which undergoes demethylation modification. Functionally, we identified that GNB4 induced oncogenic behaviors of tumors via the Hippo**–**YAP1 pathway in both in vitro and in vivo models.

**Conclusions:**

Our findings demonstrate that *H. pylori* infection activates the NF-κB-TET1-GNB4 demethylation-YAP1 axis, which may be a potential therapeutic target for GC.

**Supplementary Information:**

The online version contains supplementary material available at 10.1186/s12916-023-02842-6.

## Background

Gastric cancer (GC) is the fifth most common cancer and the fourth leading cause of cancer-related deaths globally [1]. *Helicobacter pylori* (*H. pylori*) infection is the greatest risk factor for developing GC, and the bacterium is considered a class I carcinogen by the World Health Organization [2]. However, the mechanism by which *H. pylori* induces GC has not been fully elucidated. Therefore, a better understanding of the molecular events by which *H. pylori* drives gastric tumorigenesis is required to improve current diagnostic, prognostic, and therapeutic approaches.

Aberrant promoter DNA methylation is a well-characterized mechanism of GC [3]. Several investigators have reported that altered DNA methylation patterns caused by *H. pylori* infection of gastric epithelial cells are thought to promote the risk of GC [4]. We performed a comprehensive analysis to develop DNA methylation signatures for the *H. pylori*-infected patients with GC and observed high demethylation levels of guanine nucleotide-binding protein subunit beta-4 (GNB4) [5]. GNB4 is one of the important components of heterotrimeric G proteins, which regulate the biological behavior of cells by transmitting upstream signals from G protein-coupled receptors (GPCRs) to downstream pathways [6]. GNB4 is aberrantly expressed in several tumors including GC [7–10]. However, the detailed epigenetic modification of GNB4 and the critical mediators participating in DNA demethylation in GC has not been elucidated.

DNA demethylation is mainly catalyzed by the ten–eleven translocation (TET) family of methylcytosine dioxygenases [11–13]. TETs (TET1, TET2, and TET3) catalyze the stepwise conversion of 5-methylcytosine (5mC) in DNA to 5-hydroxymethylcytosine (5hmC) and other oxidation products [14, 15]. Given that DNA methylation is relatively reversible and dynamic, activation of oncogenes or tumor suppressor genes by promoter demethylation might enhance their tumor-promoting or suppressive functions. For instance, TET1 regulated the DNA demethylation level of downstream genes or its promoter to activate them for participating in tumor progression [16–18]. Therefore, we aimed to evaluate the demethylated modulation of GNB4 by TETs in *H. pylori*-infected infection models.

Hippo pathway dysregulation is a common incident in GC, where the pathway includes two main downstream effectors, Yes-associated protein1 (YAP1) and transcriptional co-activator with PDZ-binding motif (TAZ) [19]. Recent studies have demonstrated the close relationship between *H. pylori* infection and the Hippo**–**YAP1 pathway [20–22]. YAP1 is activated and translocated to the nucleus when extracellular signaling shuts down the Hippo cascade. It binds to the TEAD family of sequence-specific transcription factors in the nucleus to initiate transcriptional expression of downstream target oncogenes, such as cysteine-rich angiogenic inducer 61 (CYR61) and connective tissue growth factor (CTGF) [23]. Furthermore, GPCR signaling regulates the Hippo**–**YAP pathway depending on the coupled G-protein [24]. To gain insights into the mechanisms by which GNB4 are linked to the malignant progression of GC, we investigated whether their expression could be linked to known tumorigenic pathways by gene set enrichment analyses (GSEA). Specifically, signatures of the Hippo–YAP1 pathway were strongly associated with high GNB4 expression. Therefore, it will be interesting and important to determine whether *H. pylori* is involved in the regulation of the Hippo**–**YAP1 pathway through GNB4.

In the present study, we found that high expression of GNB4 was significantly associated with GC progression and poor prognosis. *H. pylori* infection activated GNB4 expression and promoted the malignant phenotype of GC cells in a GNB4-dependent manner. *H. pylori* infection remarkably decreased the methylation level of the GNB4 promoter region in GC cell lines and the clinical cohort. The direct binding of TET1 to the promoter region of GNB4 induced its active demethylation, thereby activating GNB4 expression. Additionally, inhibition of YAP1 expression significantly blocked the pro-cancer function of GNB4 in *H. pylori*-induced GC models. Overall, we provided new insights into TET1-mediated GNB4 demethylation and elucidated a novel molecular mechanism underlying gastric carcinogenesis.

## Methods

### Human tissue samples

We collected 107 patient tumor samples that were pathologically diagnosed as GC from Jinan Central Hospital affiliated with Shandong First Medical University from September 2018 to November 2021. The *H. pylori* infection in patients was confirmed by a combination of pathologic diagnosis, ^13^C urea breath test or serum *H. pylori* antibody test. This study was approved by the Medical Ethics Committee of Jinan Central Hospital affiliated with Shandong First Medical University, and the patient samples were collected and processed according to the approved guidelines. Informed consent was acquired from each involved patient.

### Cell culture and reagents

Human gastric epithelial cells (GES1) and GC cell lines (MKN45, HGC27, MGC803, AGS, NCI-N87, and MKN28) were obtained from the Shanghai Institute of Biochemistry and Cell Biology, Chinese Academy of Science (Shanghai, China). AGS cells were cultured in the Ham’s F12 medium (MACGENE, Beijing, China) with 10% FBS (Gibco, CA, USA). GES1 and other GC cell lines were maintained in RPMI-1640 (MACGENE) medium supplemented with 10% FBS. All these cells were cultured at 37 °C in a humidified 5% CO_2_ incubator (Thermo Fisher Scientific, MA, USA). All cell lines were routinely tested for mycoplasma infection.

### Gene expression data analysis

The mRNA expression and clinical data of 375 GC and 32 normal control samples were obtained from The Cancer Genome Atlas (TCGA, http://cancergenome.nih.gov/) database to analyze the GNB4 expression in GC. In addition, three GC datasets with unpaired adjacent noncancer tissues (GSE13911, GSE84437, and GSE40634), a paired GC dataset (GSE65801), and *H. pylori*-infected (*Helicobacter felis* CS1 strain) C57BL/6 mouse model dataset (GSE13873) were collected from the Gene Expression Omnibus (GEO, https://www.ncbi.nlm.nih.gov/geo/) of the National Center for Biotechnology Information. The limma package in R language was used for normalization and deleting the normal or repeated samples for subsequent analysis. When a gene had multiple probes, the mean value of probes was used as the gene expression value. We also analyzed the expression information from TCGA for GNB4 using GEPIA (http://gepia.cancer-pku.cn/) [25]. Kaplan**–**Meier plotter database (http://kmplot.com/) [26] was used to verify the correlation between GNB4 expression and the prognosis of patients with GC. In addition, the CAMOIP (http://camoip.net/) tool [27] was used to screen the TCGA database for differentially expressed genes (DEGs) in patients with high and low expression of GNB4 (median value). Subsequently, the Metascape database (https://metascape.org) [28] was used for DEG enrichment analysis; |logFC| > 1, and *P* < 0.05 were considered statistically significant cutoff points.

### Gene set enrichment analysis (GSEA)

The patients of the GSE84437 dataset were divided into two groups (high and low) based on the median expression of GNB4 to analyze the GNB4-related signaling pathways. The normalization enrichment score (NES) and the false discovery rate (FDR) of each gene set were calculated using the GSEA software (v4.2.2) (http://www.gsea-msigdb.org/gsea/downloads.jsp). In addition, according to the median value of GNB4 expression, the samples were also divided into two groups in patients of the TCGA dataset, and GSEA was performed using the online tool CAMOIP (http://camoip.net/) [27]. Each gene set was considered significant when the FDR < 0.25 and |NES| > 1.

### *H. pylori* cultures and *H. pylori*-infected mouse model

*H. pylori* strains 26695 and SS1 were grown in Helicobacter Pylori Medium (Hopebio, Qingdao, China) with 7% defibrinated sheep blood (Hopebio) and *H. pylori* selective supplement (Hopebio) according to the manufacturer’s instructions under microaerobic conditions (5% O_2_, 10% CO_2_, and 85% N_2_) at 37 °C. *H. pylori* strains 26695 and SS1 were used for in vitro co-culture with gastric cancer cells or gastric epithelial cells at a multiplicity of infection (MOI) of 100:1. All mice were maintained and handled following the National Institutes of Health Guide for the Care and Use of Laboratory Animals. In addition, 48 male C57BL/6 mice (Vital River Laboratory Animal Technology, Beijing, China) were divided into four groups randomly by weight. Group 1 (control group, *n* = 18) and 2 (*N*-methyl-*N*-nitrosourea group, also named MNU group, *n* = 6) mice were treated with phosphate-buffered saline (PBS) gavage. The mice from groups 3 (SS1 group, *n* = 18) and 4 (MNU+SS1 group, *n* = 6) were infected with SS1 strain by oral gavage (1 × 10^9^ colony-forming units/mouse) for 4 months. During this period, groups 2 and 4 were also treated with MNU (5 mg MNU in 3 ml H_2_O) by gavage once a week (0.2 ml/mice for 10 weeks). Then, we randomly selected 12 mice each from groups 1 and 3 for downstream animal experiments; the remaining mice were directly executed for further analysis.

Six mice each from groups 1 and 3 were randomly selected and intraperitoneally injected with doxycycline (DOX; 30 mg/kg), and the remaining six mice were intraperitoneally injected with PBS (0.2 ml/mice) once a week for 8 weeks. The timing and order of treatment were randomized for each mouse. The outcome of the experiment was determined by pathological examination of the mouse stomach. Finally, all the mice were euthanized by inhalation of carbon dioxide. All mice with successful *H. pylori* colonization and in good condition were included in the study, except for mice that died due to complications such as gastric perforation during gavage. The flow chart of animal experiments is depicted in Fig. 5G. The mouse handling and data collection processes were participated by different investigators to ensure process blinding.

### Cell transfection and lentivirus infection

The TET1 small interfering RNA (siRNA) or respective negative control (NC) siRNA were purchased from Ribobio (Guangzhou, China). The siRNAs were transfected into cells using Lipofectamine2000 transfection reagent (Invitrogen, CA, USA) according to the manufacturer’s instructions. The GNB4-silencing lentivirus (shGNB4), negative control lentivirus (shControl), GNB4-overexpressing lentivirus (GNB4), and blank control lentivirus vector (Vector) were constructed by GENECHEM (Shanghai, China). The corresponding sequence information is listed in Additional file 9: Table S1. GC cells were seeded in 24-well plates, grown overnight, and then infected with lentivirus. The lentivirus infection rate was measured as the percent of cells expressing GFP fluorescence 72 h following infection. In addition, we added puromycin (2 µg/ml; Sigma-Aldrich, Missouri, USA) to the culture medium for at least 1 week.

### RNA isolation and real-time quantitative polymerase chain reaction (qRT-PCR)

Total RNA was extracted using TRIzol Reagents (Invitrogen). Total RNA was reverse-transcribed to cDNA using a HiFiScript gDNA removal cDNA synthesis kit (CWBIO, Taizhou, China). qRT-PCR was performed using the UltraSYBR Mixture (Low ROX; CWBIO) on a LightCycler 480 Real-Time PCR System (Roche, Basel, Switzerland). The sequences of the primers are listed in Additional file 9: Table S2.

### Immunohistochemistry (IHC)

Paraffin-embedded tissues were sectioned, dewaxed, and subjected to high temperature-antigen retrieval in EDTA antigenic-retrieval buffer for 20 min to shelter endogenous peroxidase activity. IHC assays were performed using a biotin assay (Beijing Zhongshan Jinqiao, China) following the manufacturer’s instructions. The samples were incubated with anti-GNB4 primary antibody (1:200; MyBioSource, CA, USA) overnight at 4 °C. Next, the sections were incubated with corresponding secondary antibody and finally analyzed using a DAB staining kit (Solarbio, Beijing, China). The intensity of positive staining was scored by “IHC score” as follows: 0 (negative), 1 (weak), 2 (moderate), and 3 (strong). The proportion of positively stained cells was scored as 0 (0%), 1 (< 25%), 2 (25–75%), and 3 (> 75%). The IHC score was obtained by multiplying the above two scores.

### Western blotting

Total proteins were extracted using RIPA lysis buffer (Beyotime, Beijing, China); nuclear and cytoplasmic proteins were extracted using the Nuclear and Cytoplasmic Protein Extraction Kit (Beyotime) containing protease inhibitors. Protein concentration was measured using a BCA Protein Assay Kit (Beyotime). The proteins were separated using SDS-PAGE and then transferred to the PVDF membranes (Millipore, MA, USA). The blotted membranes were blocked with 5% non-fat milk for 2 h at room temperature (20 ℃ ± 5 ℃) and incubated with primary antibody overnight at 4 °C. The details of primary antibodies and the respective dilutions used in this study are included in Additional file 9: Table S3. Next, the membranes were incubated with secondary antibodies, and the bands were detected using an ECL detection reagent (Millipore).

### Immunofluorescence assay

Cells subjected to different treatments were harvested, fixed with 4% paraformaldehyde for cell sides, and permeabilized with 0.5% Triton X-100 (Solarbio). After washing and blocking, the cells were incubated overnight at 4 °C with anti-GNB4 (1:250; Proteintech Group, Wuhan, China) and *H. pylori* anti-CagA (1:300; GeneTex, TX, USA) antibodies. After washing with PBS (3×), cells were incubated with 1:400 Alexa Fluor-647 or Alexa Fluor-546 (Thermo Fisher, FL, USA) secondary antibody for 1 h in the dark. Finally, the nuclei were stained with 40,6-diamidino-2-phenylindole (DAPI) (Solarbio), and a confocal laser scanning microscope (LeicaSP8, Germany) was used to acquire the images.

### Cell proliferation and colony formation assays

Cell proliferation was also assayed using Cell Counting Kit-8 (CCK-8; Dojindo, Japan). Briefly, the cells were seeded into 96-well plates and incubated with the indicated treatments. Subsequently, we added 100 µl of fresh medium to cells containing 10 µl of CCK-8 solution and incubated the plate for 2 h (37 °C, 5% CO_2_). Next, the absorbance was measured at 450 nm using a spectrophotometer; the recorded absorbance values were normalized to the absorbance of blank wells. Cell viability was assessed by trypan blue staining. In short, after 3, 6, and 9 h of *H. pylori* infection of MKN45 cells inoculated in 6-cm dishes and incubated for 72 h, we performed live cell counts using Countstar® (Countstar Inc.), an automated cell counter (mix 1:1 volume ratio of 0.2% trypan blue to PBS before counting).

For colony formation assays, the cells (500 cells/well) were seeded in a 6-well plate and incubated for 1–2 weeks until the colonies appeared. Colonies were counted after fixing with methanol followed by staining with 0.5% crystal violet.

### Ethynyldeoxyuridine (EdU) staining

The percentage of DNA-replicating cells representing the proliferative state of the cells was determined using the Cell-Light EdU Apollo567 In Vitro Kit (RiboBio, Guangzhou, China) according to the manufacturer’s instructions.

### Wound healing and transwell assays

For the wound healing assays, 2 × 10^5^ cells were seeded into a 24-well plate until confluence. The cell monolayer was scratched using a pipette tip. After 48 or 72 h, the wound area was measured, and the percentage of the wound closure was calculated as (wound area at 0 h − wound area at 48 h or 72 h)/wound area at 0 h × 100. The wounds were imaged using an inverted phase-contrast microscope.

For transwell assays, 5 × 10^4^ cells were suspended in 200 μl serum-free medium and seeded into a 24-well Boyden chamber (8-μm pore size; Corning, NY, USA) with Matrigel (BD Biosciences; NJ, USA) to detect cell invasion ability or without Matrigel to detect cell migration ability. The lower chamber was supplemented with a medium containing 20% FBS. After 48 or 72 h, cells affixed to the lower surface of the chambers were fixed with 4% paraformaldehyde, stained with 0.1% crystal violet, and counted under the microscope.

### Methylation-specific PCR (MSP), pyrosequencing, and mass array methylation detection

Total DNA was extracted from cells and tissues using Universal Column Genome Extraction Kit (CWBIO). For the MSP assay, 30 ng of the extracted DNA was subjected to bisulfite conversion using an EZ DNA Methylation-Gold™ kit (Zymo Research, CA, USA) according to the manufacturer’s instructions. Bisulfite-converted DNA was then used as a template for PCR using EpiScope MSP Kit (TaKaRa, Japan); PCR was performed using the standard manufacturer’s protocol. The primer sequences for the MSP are shown in Additional file 9: Table S4. The PCR products were separated using 3% agarose gel.

Pyrosequencing and mass array methylation detection were performed to quantitatively evaluate the DNA methylation level of CpG sites in the GNB4 promoter. First, the bisulfite conversion of extracted DNA was performed according to the manufacturer’s instructions. For pyrosequencing, a specific GNB4 promoter region (chr3:179451774–179451745, GRCh38.p13 version) was amplified from bisulfite-treated genomic DNA using primers designed via PyroMark Assay Design 2.0 (Additional file 9: Table S4). The PCR products were turned into pyrosequencing using the PyroMark Q96 pyrosequencing and quantification platform following the manufacturer’s instructions (OE Biotech Co., Ltd., Shanghai, China). Similarly, PCR primers (Additional file 9: Table S4) were designed using EpiDesigner for mass array methylation detection. Small RNA fragments with CpG sites were acquired from the PCR products by RNaseA digestion. CpG methylation was detected using the MassARRAY platform, and the methylation rate was calculated by the Epityper software version 1.0 (Agena, San Diego, CA, USA). The detection of methylation and subsequent analysis were performed by the OE Biotech Co., Ltd.

### Chromatin immunoprecipitation (ChIP) assay

ChIP assay was conducted using the SimpleChIP® Enzymatic Chromatin IP Kit (Cell Signaling Technology, MA, USA) according to the manufacturer’s protocol. In brief, GC cells were cross-linked in PBS containing 1% formaldehyde at room temperature for 10 min. Subsequently, we destroyed the cross-linked chromatin DNA using sonication. The chromatin was then immunoprecipitated using an anti-TET1 antibody (Active Motif Inc, CA, USA). Normal rabbit IgG was used as the negative control. Finally, precipitated DNA was purified and then analyzed by using qPCR. Primers for ChIP-qPCR are listed in Additional file 9: Table S5.

### Co-immunoprecipitation (Co-IP)

The co-IP assay was performed using the Pierce® immunoprecipitation kit (Thermo Fisher Scientific) according to the manufacturer’s instructions. Briefly, AGS cells were lysed by ice-cold IP lysis/wash buffer and centrifuged at 13,000×*g* for 10 min to remove the debris. The supernatant was further immunoprecipitated with an anti-YAP1 antibody (1:50; Cell Signaling Technology). Rabbit IgG was used as a negative control. Precipitates were separated using SDS-PAGE and further analyzed by performing immunoblotting.

### Animal studies

We established subcutaneous GC animal xenograft models to assess carcinogenicity in vivo. Stable GNB4-overexpressing GC cells (4 × 10^6^) and control cells were subcutaneously injected into the left flanks of 24 5-week-old BALB/c nude mice (Vital River Laboratory Animal Technology, Beijing, China; *n* = 24/12 in each group). In addition, six mice each were randomly selected by weight from the GNB4-overexpression and control groups for simultaneous intraperitoneal injection of verteporfin (VP; 100 mg/kg, qd, a week). Tumor growth was monitored every 3 days and the mice were culled after 21 days. The tumor volume was calculated as volume = (width^2^ × length)/2. To reduce the interference of confounding factors in data collection, tumor size was measured from 9 a.m. to 9 p.m. The order and time of measurement were randomized for each mouse. Furthermore, we developed tail vein metastasis models to assess the metastatic properties. Stable GNB4-overexpressing MKN45 and HGC27 cells (1 × 10^6^) and the control cells were injected into the tail vein of 24 nude mice. Similarly, 3 weeks later, six mice each were selected by weight in the GNB4-overexpression and control groups for simultaneous intraperitoneal injection of VP (100 mg/kg, qd, a week). In case of complications such as pulmonary embolism leading to death, these mice were excluded from the study and the rest of the mice were included in the study. After 6 weeks of normal feeding, the mice were euthanized by inhalation of carbon dioxide and dissected to observe their liver and lung tumor metastatic nodules. The liver and lungs of the mice were dehydrated, fixed, and subjected to hematoxylin and eosin (H&E) staining (Solarbio) according to the manufacturer’s instructions. The mouse handling and data collection processes were participated by different investigators to ensure process blinding.

### Statistical analysis

The GraphPad Prism 8.0 and R software (v4.1.3) were used for statistical analysis. Experiments were independently repeated at least three times. Student’s *t*-test one-way ANOVA were used to determine the significance of two groups and multiple groups, respectively. Two-way ANOVA was used to analyze the differences between the two groups over time. Spearman correlation was used to determine the expression correlation of two genes. ROC curves were plotted and statistically analyzed using the MedCalc software (v18.2.1). A chi-square test was used to determine the relationship between GNB4 and clinicopathological variables. The sample size of mice was estimated by the degrees of freedom of ANOVA. Data were presented as mean ± standard deviation.* P* < 0.05 was considered statistically significant (ns, not significant, *P < 0.05, **P < 0.01, ***P < 0.001, and ****P < 0.0001).

## Results

### GNB4 is highly expressed in GC and is involved in the malignant process

**Fig. 1:**
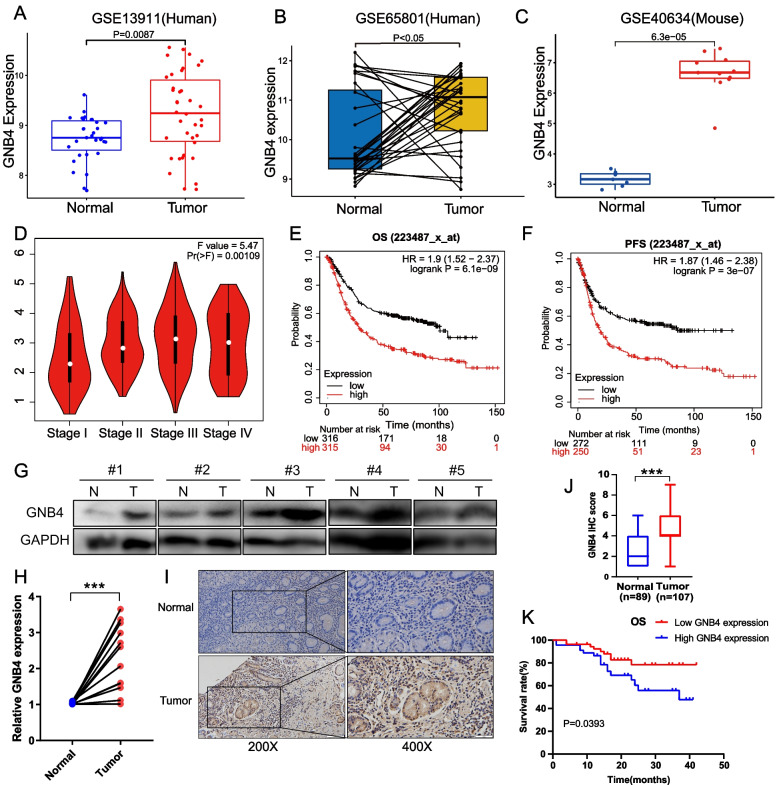
GNB4 is upregulated in GC. **A** GNB4 expression is significantly upregulated in GC tissues (*n* = 31) compared with that in normal tissues (*n* = 38) from the GEO dataset. **B** The mRNA expression levels of GNB4 are examined in the paired tumor and adjacent nontumor tissues (*n* = 32 pairs) from the GEO dataset. **C** The mRNA expression of GNB4 is significantly upregulated in tumor tissues of the mouse GC model compare to normal tissues (GSE40634). **D** GEPIA analysis shows that GNB4 expression is significantly correlated with the clinical staging of patients with GC. **E**, **F** Kaplan–Meier plotter analysis reveals that patients with higher GNB4 expression have worse overall survival (OS, **E**) and progression-free survival (PFS, **F**) compared with patients with lower GNB4 expression. **G** Protein expression of GNB4 in GC tissues (T) and adjacent normal tissues (N). **H** qRT-PCR analysis of GNB4 mRNA expression in paired GC tissues and adjacent normal tissues (*n* = 10). **I**, **J** IHC staining (**I**) and H-score (**J**) for GNB4 in adjacent normal tissues (*n* = 89) and GC tissues (*n* = 107). Scale bars: 100 μm (inset: 50 μm). **K** Overall survival was analyzed using Kaplan–Meier curves (log-rank test) in a GC cohort of 101 patients. GC, gastric cancer

We analyzed the expression of GNB4 in human GC patient samples from the GEO database. GNB4 expression was elevated in GC tissues in both unpaired (GSE13911, *P* < 0.01, Fig. 1A) and paired GC sample datasets (GSE65801, *P* < 0.05, Fig. 1B). Moreover, analysis of stomach tissues in the public data set of Prom1-conditional mutant mouse GC model from the GEO database (GSE40634) demonstrated significant overexpression of GNB4 mRNA in the mouse tumor tissues (*P* < 0.0001, Fig. 1C). In addition, GEPIA analysis revealed that the GNB4 expression increased with the progression of clinical staging (*P* < 0.01, Fig. 1D). Furthermore, receiver operator characteristic (ROC) curves indicated that GNB4 expression could be applied to distinguish patients with GC with or without lymph node metastasis (Additional file 1: Fig. S1A) with an area under the ROC curve (AUC) of 0.576 (*P* < 0.05). We determined the prognostic value of GNB4 in GC using the publicly accessible Kaplan–Meier plotter online platform and found that patients with GC having a high GNB4 expression exhibited shorter overall survival (OS; *P* < 0.0001, Fig. 1E) and progression-free survival (*P* < 0.0001, Fig. 1F).

We further validated the GNB4 expression status and clinical association with our own clinical GC tissue cohort. Our analysis also suggested that the expression of GNB4 in the GC tissue samples was higher than that in the normal tissues (*P* < 0.05, Fig. 1G–J). Consistent with the above results, Kaplan–Meier survival curves showed that patients with high GNB4 expression displayed adverse OS (*P* < 0.05, Fig. 1K) in our cohort. We also performed qRT-PCR and western blotting in six GC cell lines and one normal gastric epithelial cell line (GES1). Our data indicated that the GNB4 mRNA and protein expression was significantly higher in GC cells than in GES1 cells (*P* < 0.01, Additional file 1: Fig. S1B, C). These results suggest that GNB4 may play a crucial role in gastric carcinogenesis.

### *H. pylori* infection induces GNB4 expression in vitro and in vivo

**Fig. 2:**
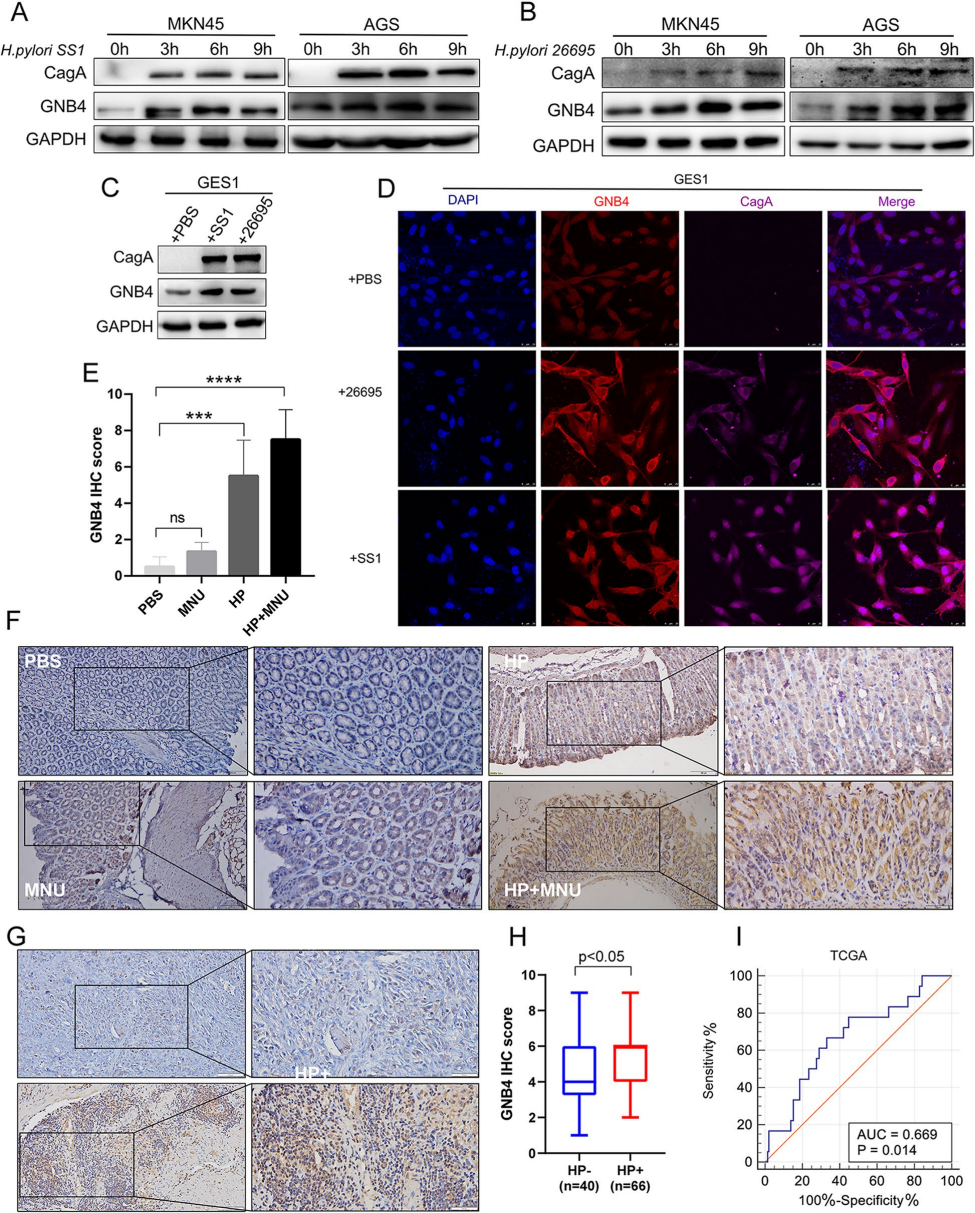
*H. pylori* infection induces GNB4 overexpression in vitro and in vivo. **A**, **B** Western blot analysis of GNB4 and CagA protein expression in AGS and MKN45 cells infected with *H. pylori* SS1 (**A**) and *H. pylori* 26695 (**B**) at different time points. **C** Western blot analysis of GNB4 protein expression in GES1 cells infected with *H. pylori* 26695 or *H. pylori* SS1 for 6 h. **D** GES1 cells were infected with *H. pylori* 26695 and *H. pylori* SS1 for 6 h. Representative images of immunofluorescence staining (scale bars: 25 µm) for GNB4 (red), CagA (purple), and DAPI (blue). **E**, **F** IHC score (**E**) and IHC staining (**F**) of GNB4 in the PBS group (*n* = 6), HP (SS1 strain)-infected (*n* = 6), MNU (*n* = 6), and HP+MNU (*n* = 6) mice. Scale bars: 100 μm (inset: 50 μm). **G**, **H** IHC staining (**G**) and IHC score (**H**) of GNB4 in *H. pylori*-negative (HP−; *n* = 40) and *H. pylori*-positive (HP+; *n* = 66) patients with GC. **I** Area under the receiver operator characteristic (ROC) curve for GNB4 in the differential diagnosis of GC with and without *H. pylori* infection (AUC = 0.6614; *P* = 0.014). Scale bar: 100 μm (inset: 50 μm). HP, *Helicobacter pylori*; IHC, immunohistochemistry; MNU, *N*-methyl-*N*-nitrosourea; AUC, area under curve

*H. pylori* infection is considered a high-risk factor for GC [29]. Therefore, we conducted in vitro and in vivo experiments to further verify whether *H. pylori* infection plays an important role in regulating GNB4 expression. First, we infected MKN45 and AGS cells with *H. pylori* strains (SS1 and 26695) at different time points. Western blot analysis showed that *H. pylori* infection significantly upregulated GNB4 protein levels in the human GC cell lines, and the upregulation was highest at 6 h post-infection (Fig. 2A, B). Then, qRT-PCR results showed that GNB4 mRNA expression was significantly induced in GC cell lines after 6 h of infection with *H. pylori* strains (*P* < 0.001, Additional file 2: Fig. 2A). Notably, similar results were obtained using GES1 cells (Fig. 2C). In addition, immunofluorescence staining demonstrated a significant increase in GNB4 expression and cytoplasmic fluorescence intensity 6 h after inducing *H. pylori* infection in GES1 and AGS cells (*P* < 0.0001, Fig. 2D, Additional file 2: Fig. S2B, C). A previous study has reported that *H. pylori* gavage promotes the progression of gastric precancerous lesions in mice [30]. Next, we infected mice with *H. pylori* strain SS1, intraperitoneally injected MNU for 4 months (Fig. 5G), and examined GNB4 expression levels. IHC analysis revealed that *H. pylori*-infected group showed significantly upregulated GNB4 expression in the gastric mucosa compared with the MNU group. The GNB4 protein level was the highest in the *H. pylori* combined with the MNU group (Fig. 2E, F). We also included a public dataset of *H pylori*-infected (Helicobacter *felis* CS1 strain) C57BL/6 mouse model from GEO (GSE13873) and found that GNB4 mRNA levels were also significantly upregulated in *Helicobacter*-infected gastric mucosa (*P* < 0.05, Additional file 2: Fig. 2D). Furthermore, the IHC analysis of human GC tissues from our cohort showed high levels of GNB4 protein in *H. pylori*-positive (HP+) tumor tissue samples compared to those in *H. pylori*-negative (HP−) samples (*P* < 0.05, Fig. 2G, H). In addition, increased GNB4 expression was significantly correlated with the TNM-T and clinical stages in HP+ patients with GC (Table 1). Notably, GNB4 expression could be used to differentiate patients with GC with or without *H. pylori* infection in the TCGA database (Fig. 2I; AUC = 0.669, *P =* 0.014). These findings indicated that *H. pylori* infection played an important role in the induction of GNB4 expression.

### *H. pylori* promotes GC cells proliferation, migration, and invasion via GNB4

**Fig. 3:**
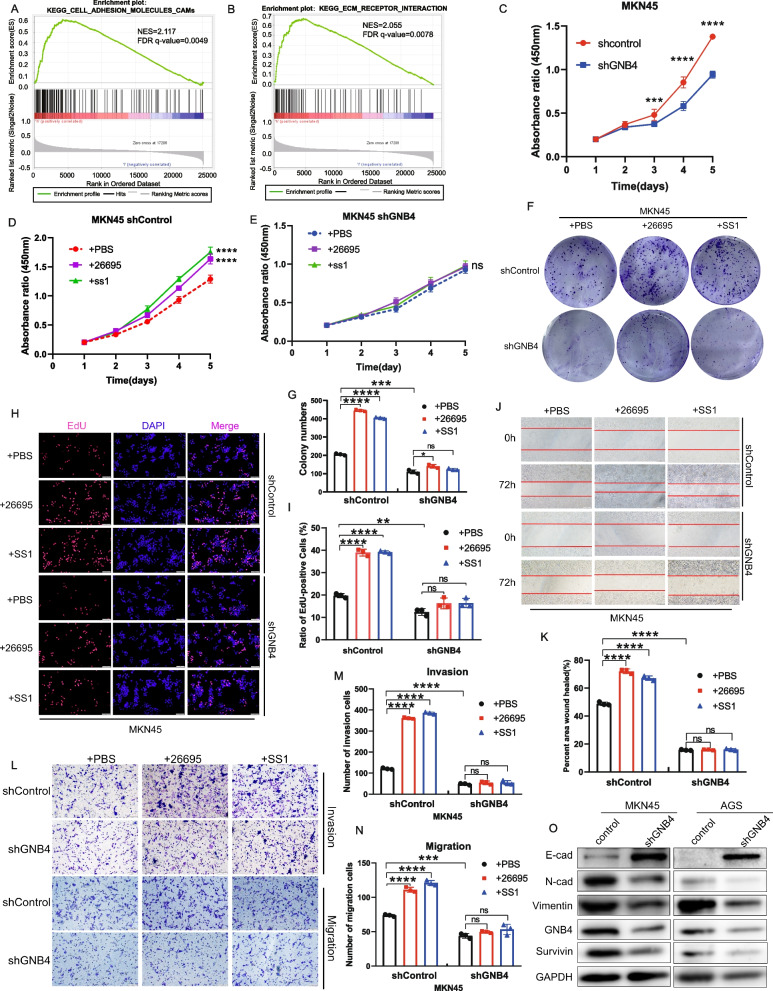
*H. pylori* triggers malignancy in MKN45 cells via regulating the GNB4 expression. **A**, **B** Gene set enrichment analysis (GSEA) based on gene expression profiling of patients with GC (*n* = 433) in the GSE84437. GNB4 significantly correlates with cell adhesion molecules (CAMs) (**A**) and ECM-receptor interaction pathway (**B**). **C**–**E** CCK-8 assay was performed to monitor the cell proliferation in MKN45 shControl or shGNB4 (**C**) and changes in proliferation of MKN45 shControl (**D**) and shGNB4 (**E**) uninfected or infected with *H. pylori* strains (26695 and SS1; 6 h). **F**, **G** Colony formation assays were performed to evaluate the proliferation abilities of MKN45 shControl and shGNB4 uninfected or infected with *H. pylori* strains (26695 and SS1; 6 h). Representative images (**F**) and histograms presenting the colony numbers in each group (**G**) are shown. **H**, **I** EdU assays were conducted in MKN45 shControl and shGNB4 uninfected or infected with *H. pylori* strains (26695 and SS1; 6 h) to compare the percentage of cells in the S phase (scale bar: 100 μm). DAPI staining detected total cells, whereas EdU staining represented cells with active DNA replication. Representative images (**H**) and quantification data (**I**) are shown. **J**, **K**. Wound healing assays were conducted to compare the migration capabilities of MKN45 shControl and shGNB4 uninfected or infected with *H. pylori* strains (26695 and SS1; 6 h). The difference in cell margin at 0 and 72 h showed the moving track of cells (scale bar: 200 μm) (**J**), and the percentage of the healed area was quantified (**K**). **L**–**N** Transwell assays of MKN45 shControl and shGNB4 uninfected or infected with *H. pylori* strains (26695 and SS1; 6 h) were performed to measure the migration and invasion abilities of cells (scale bars: 100 μm). Bar charts show the number of cells which passed through the chamber membrane in each group (**M**, **N**). Data are presented as the mean ± SD of three independent experiments. **O** Protein expression of GNB4, epithelial marker E-cadherin, mesenchymal markers (N-cadherin and vimentin), and survivin in control and shGNB4-expressing GC cells

We performed GSEA to verify the functional value of GNB4 expression; GSEA was based on the gene expression profile in a public set of 433 patients with GC from GEO (GSE84437). The high-GNB4-expression group was markedly enriched in epithelial-to-mesenchymal transition (EMT)-related pathways such as cell adhesion and extracellular (ECM)-receptor interaction (Fig 3A, B) and inflammation-related pathways such as chemokine signaling pathway and leukocyte transendothelial migration (Additional file 3: Fig. S3), which confirmed the close relationship between GNB4 and *H. pylori* infection. Previous studies demonstrated that GNB4 promoted the proliferation and metastasis of GC cells [9]. Given that the levels of GNB4 correlated with EMT and *H. pylori* infection, we investigated whether *H. pylori* could exert its cancer-promoting effects through GNB4. The results of the CCK-8 assay indicated that GNB4 knockdown significantly decreased the proliferation of MKN45 and AGS cells (*P* < 0.0001, Fig. 3C, Additional file 4: Fig. S4A). The proliferation rates of shControl cells were significantly increased after *H. pylori* 26695 and SS1 infection (*P* < 0.0001, Fig. 3D, Additional file 4: Fig. S4B). Notably, GNB4 knockdown blocked the promotion effects of *H. pylori* infection on cell proliferation (Fig. 3E, Additional file 4: Fig. S4C). We compared the proliferation ability of *H. pylori*-infected MKN45 cells at 3, 6, and 9 h, and live cell counts revealed that MKN45 cells proliferated most strongly at 6 h of infection (Additional file 4: Fig. S4D, E). Furthermore, colony formation and EdU assays similarly showed that the knockdown of GNB4 in GC cells inhibited the pro-proliferative ability of *H. pylori*. (*P* < 0.01, Fig. 3F–I, Additional file 4: Fig. S4F, G). Furthermore, wound healing and transwell assays showed that the migration and invasive abilities of MKN45 and AGS cells were enhanced after infection with *H. pylori* strain (26695 or SS1) infection; however, GNB4 knockdown diminished these abilities (*P* < 0.001, Fig. 3J–N, Additional file 4: Fig. S4H-L). Next, we performed western blotting to detect the expression of EMT and cell proliferation regulatory proteins in GC cell lines. Our data showed that GNB4 knockdown significantly decreased the expression of N-cadherin, vimentin, and survivin in GC cell lines; however, it increased the expression of E-cadherin (Fig. 3O). Collectively, these results indicated that *H. pylori* infection may contribute to the proliferation, migration, and invasion of GC cells in a GNB4-dependent manner.

### *H. pylori* infection induces demethylation modification of the GNB4 promoter region

**Fig. 4:**
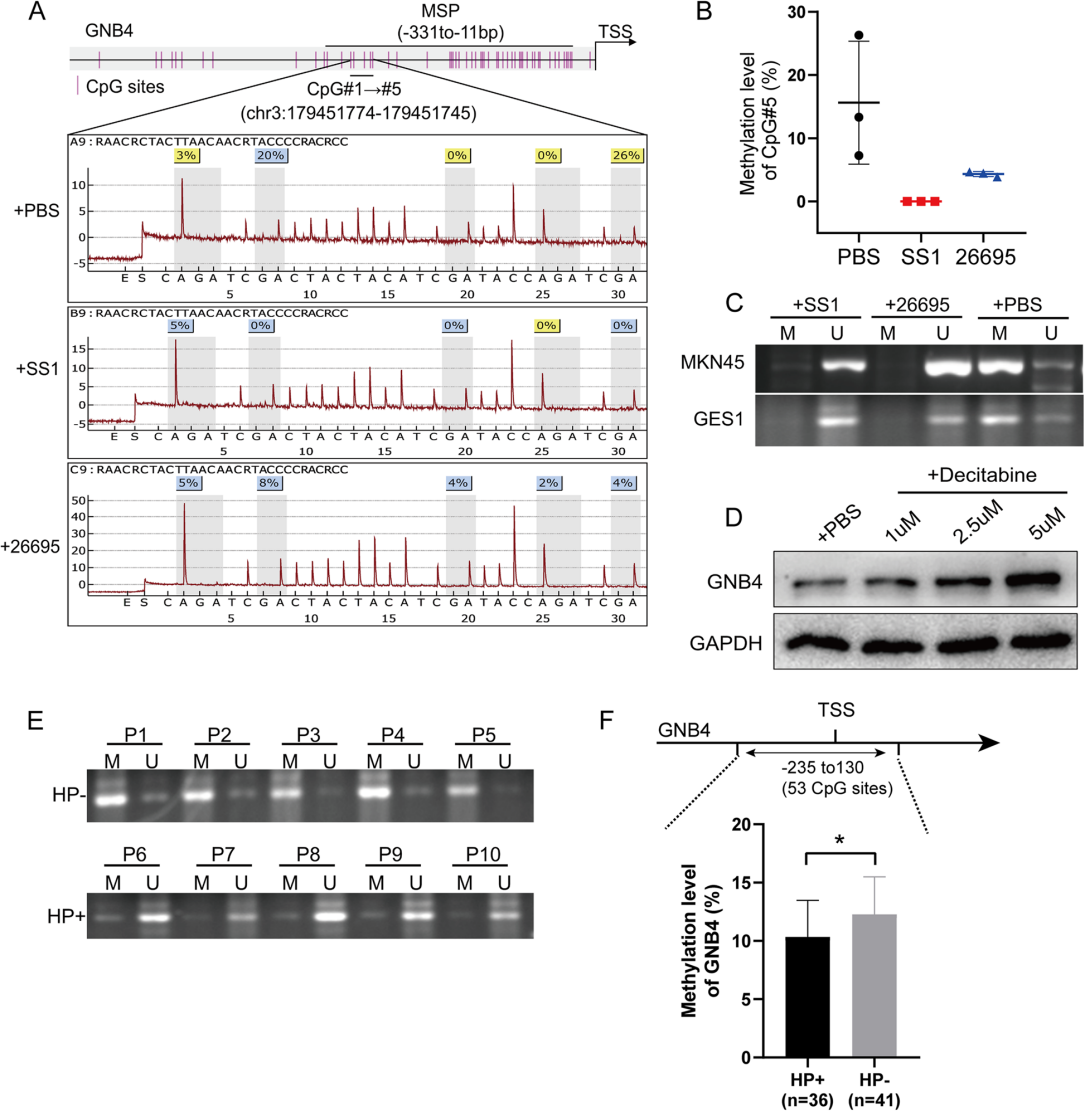
*H. pylori* infection induces demethylation modification of the promoter region of GNB4. **A** Pyrophosphate sequencing of the promoter region of GNB4 (5 CpG sites, chr3:179451774–179451745) in MKN45 cells uninfected or infected with *H. pylori* strains (26695 and SS1) for 6 h. The upper part of the panel shows the primer sequence regions for methylation-specific PCR (MSP). The bottom part of the panel shows a representative image of the results of pyrophosphate sequencing. **B** Methylation levels of the CpG#5 site (chr3:179451746–179451745) in three independent experiments via pyrophosphate sequencing. **C** MSP analysis showed GNB4 promoter region undergoes decreased methylation levels after *H. pylori* infection (26695 and SS1; 6 h) in MKN45 and GES1 cells. M, methylation-specific PCR product; U, unmethylation-specific PCR product. **D** Western blot analysis of GNB4 in MKN45 cells treated with different concentrations of decitabine (DNA methylation inhibitor) for 24 h. **E** Compared to *H. pylori*-negative (HP−) GC samples, the promoter methylation level of GNB4 in *H. pylori*-positive (HP+) GC samples was significantly decreased (verified by MSP). **F** Mass spectrometry-based analysis of the difference in methylation levels of the indicated CpG island region (53 CpG sites; − 235 to 130 bp; location of the GNB4 promoter) between HP+ and HP− patients with GC

Chronic *H. pylori* infection induces abnormal methylation accumulation in the gastric mucosa [4]. In addition, our previous bioinformatic analysis revealed that the GNB4 expression was negatively correlated to its methylation level [5]. Therefore, we investigated whether methylation modification is the central mechanism triggering GNB4-induced proliferation and metastasis of gastric cancer cells. First, we performed pyrophosphate sequencing of the GNB4 promoter region. The results showed that *H. pylori* infection reduced the methylation level of CpG#5 (chr3:179451746–179451745) but not of the other four CpG sites in GC cells (Fig. 4A, B, Additional file 5: Fig. S5). Next, we designed specific primers based on the CpG island (containing the differentially methylated site CpG#5), performed MSP, and found that *H. pylori* infection significantly reduced the methylation level of the GNB4 promoter region in MKN45 cells (Fig. 4C). Furthermore, the expression of GNB4 was upregulated in a dose-dependent manner, when decitabine (DNA methyltransferase inhibitor) was added to MKN45 cells, indicating that methylation of GNB4 was involved in the regulation of its expression (Fig. 4D). Furthermore, to validate our results of in vitro experiments, MSP analysis of *H. pylori*-negative (HP−, *n* = 5) and *H. pylori*-positive (HP+, *n* = 5) GC samples from our cohort demonstrated significantly decreased methylation levels in the GNB4 promoter region in HP+ GC samples (Fig. 4E). Consistent with the MSP results, mass spectrometry also determined that the methylation level of the GNB4 promoter region was significantly reduced in HP+ GC tissue samples (*n* = 36) compared with that in HP− tissue samples (*n* = 41) (*P* < 0.05, Fig. 4F). Taken together, *H. pylori* infection induced demethylation modification of the GNB4 promoter region in human GC cell lines and clinical tissue samples.

### *H. pylori* infection induces inflammation to upregulate GNB4 expression via TET1-mediated DNA demethylation modifications

**Fig. 5:**
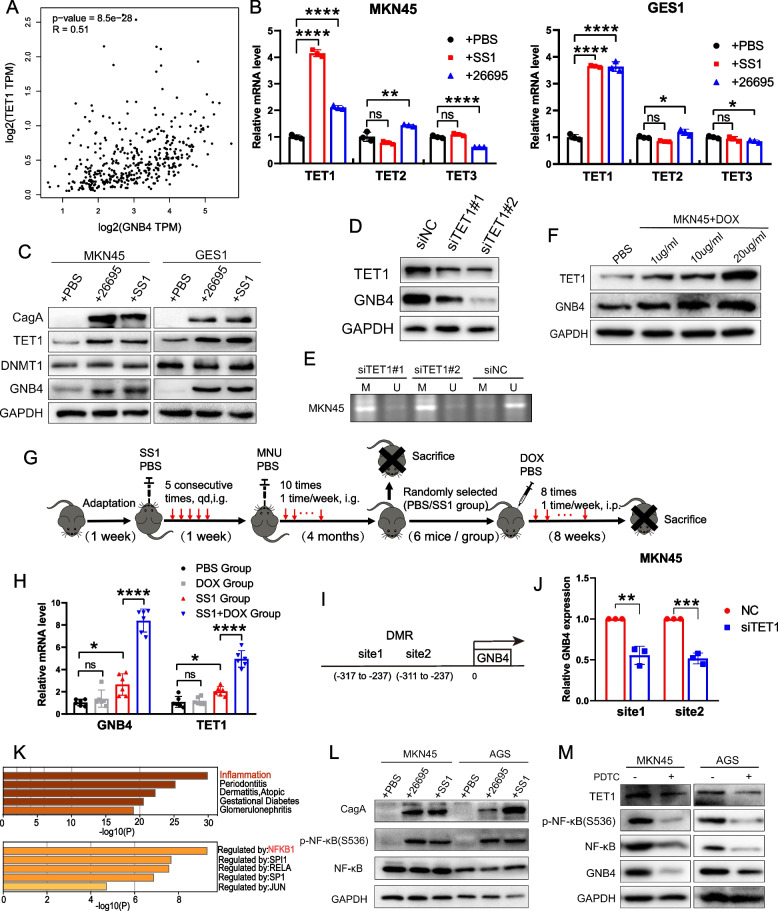
*H. pylori* regulates the GNB4 expression and demethylation modification through induction of TET1 expression. **A** GEPIA analysis revealed that GNB4 expression was significantly correlated with TET1 in patients with GC (Spearman method, *R* = 0.51, *P* < 0.001). **B** qRT-PCR analysis of TET1, TET2, and TET3 mRNA expression in GES1 and MKN45 cells uninfected or infected with *H. pylori* strains (26695 and SS1; 6 h). **C** The expression of CagA, TET1, DNMT1, and GNB4 proteins in GES1 and MKN45 cells uninfected or infected with *H. pylori* strains (26695 and SS1; 6 h) was determined through western blotting. **D** Western blot analysis of TET1 expression in MKN45 cells treated with TET1 siRNA. **E** Promoter methylation level of GNB4 in MKN45 cells treated TET1 siRNA through MSP. **F** Western blot analysis of TET1 and GNB4 expression in MKN45 cells following treatment with different concentrations of doxycycline (DOX) for 24 h. **G** Schematic description of the experimental design for establishing the animal model. **H** qRT-PCR analysis of GNB4 and TET1 expression in the gastric mucosa of different groups of mice. **I** Pattern diagram showing the two primer amplification regions designed in the differentially methylated region (DMR, − 331 to − 11 bp) of GNB4 promoter in ChIP-qPCR. **J** ChIP-PCR analysis of the GNB4 expression in MKN45 cells transfected with NC and TET1 siRNA to examine the binding of TET1 and the DMR of GNB4 promoter. **K** Five enriched diseases and regulated genes according to differential GNB4 expression in the TCGA database (using Metascape online tool). **L** Western blot analysis of CagA, p-NF-κB(S536), and NF-κB protein expression in AGS and MKN45 cells uninfected or infected with *H. pylori* strains (26695 and SS1; 6 h). **M** Western blot analysis of TET1, p-NF-κB(S536), NF-κB, and GNB4 protein expression in AGS and MKN45 cells treated with PDTC (NF-κB inhibitor; 10 and 20 μM for AGS and MKN45 cells, respectively) for 12 h

DNA methylation is regulated by DNA methylation transferases (DNMTs) [11]. When DNMT1 fails to replicate the 5mC mark, passive demethylation may occur during cell division [11, 31]. In addition, the TET proteins (TET1, TET2, and TET3) are capable of initiating active DNA demethylation pathways [14, 15, 32]. We analyzed the correlation between DNMTs or TETs and GNB4 expression in GC to search for methylation or demethylation enzymes regulating GNB4 expression using the GEPIA online database. Only TET1 showed the most significant correlation with GNB4 in terms of expression (*R* = 0.51,* P* < 0.001, Fig. 5A; Additional file 6: Fig. S6A). Furthermore, the qRT-PCR analysis demonstrated a significant upregulation of TET1 but not TET2 and TET3 mRNA expression in normal gastric epithelium and GC cells after *H. pylori* (26695 and SS1) infection (*P* < 0.0001, Fig. 5B). Consistently, the TET1 protein expression was also significantly increased after *H. pylori* infection. In contrast, no significant change was observed in DNMT1 mRNA or protein expression, suggesting that *H. pylori* may regulate GNB4 expression by affecting the active demethylation process catalyzed by TET proteins (Fig. 5C). Next, we knocked down the expression of GNB4 and TET1 in MKN45 cells to determine whether TET1 regulates the expression and methylation levels of GNB4 (Fig. 5D, Additional file 6: Fig. S6B). Our results indicated that knocked down TET1 decreased the expression of GNB4, whereas the TET1 protein expression was nearly unchanged when GNB4 was knocked down in MKN45 cells (Fig. 5D, Additional file 6: Fig. S6B). Of note, MSP analysis demonstrated that knockdown of TET1 resulted in increased methylation in the GNB4 promoter region (Fig. 5E). In line with the above results, GNB4 protein expression was also enhanced after activation of TET1 with an increasing dose of DOX, an agonist of TET1 (Fig. 5F). Moreover, the qRT-PCR analysis revealed that DOX combined with *H. pylori* infection significantly increased GNB4 expression compared to *H. pylori* infection alone, which further indicated that TET1 could induce GNB4 expression in vivo (*P* < 0.0001, Fig. 5G, H). In addition, pre-pyrophosphate sequencing results indicated two TET1-binding sites were identified in the differentially methylated region (DMR) of the GNB4 promoter region (Fig. 5I). The results of ChIP-PCR assay also showed that TET1 could directly accumulate at DMR of GNB4 promoter to affect its expression (*P* < 0.01, Fig. 5J).

*H. pylori* infection correlates with a strong oncogenic inflammatory response during gastric carcinogenesis [33]. Nuclear factor kappa B (NF-κB), a crucial determinant for chronic inflammation, could also be activated by *H. pylori* virulence factor CagA [34]. Metascape [28] analysis revealed that inflammation and NFKB1 are the first enriched disease and gene, respectively, according to differential GNB4 expression in the TCGA database (Fig. 5K). In addition, the western blot assay indicated that *H. pylori* infection significantly upregulated p-NF-κB (S536) protein levels in human GC cell lines (Fig. 5L). Moreover, an NF-κB inhibitor, pyrrolidinedithiocarbamic acid (PDTC), repressed GNB4 and TET1 expression in MKN45 and AGS cells (Fig. 5M). Collectively, these results suggested that *H. pylori* activated its expression by inducing inflammatory responses involved in TET1-mediated GNB4 demethylation modifications.

### GNB4 promotes YAP1 nuclear accumulation and its downstream target gene expression in the Hippo pathway

**Fig. 6:**
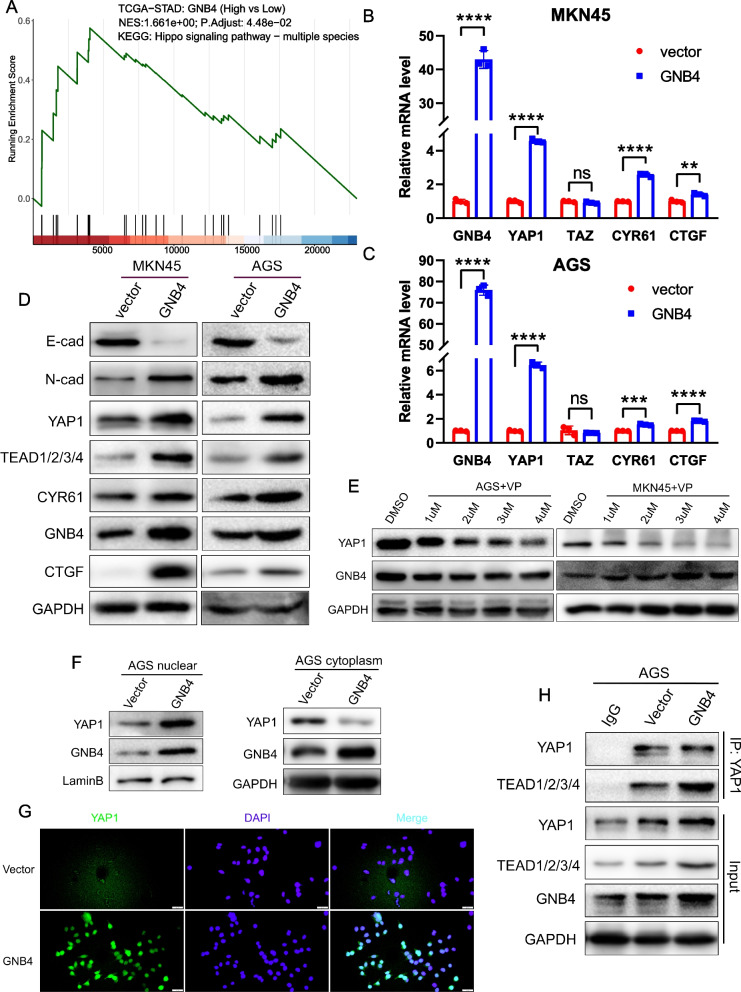
GNB4 activates downstream Hippo-YAP1 pathway molecules in GC cells. **A** GSEA was based on GNB4 expression in patients with GC (data obtained from the TCGA database) using CAMOIP online tool. **B**, **C** qRT-PCR analysis of GNB4, YAP1, TAZ, CYR61, and CTGF expression in MKN45 (**B**) and AGS (**C**) cells with or without GNB4 expression. **D** Expression of E-cadherin (E-cad), N-cadherin (N-cad), and the indicated Hippo pathway molecules (YAP1, TEAD1/2/3/4, CYR61, and CTGF) in AGS and MKN45 cells without and with GNB4 overexpression (measured by using western blotting). **E** Expression of GNB4 and YAP1 in MKN45 and AGS cells treated with increasing concentrations of verteporfin (VP) for 12 h (using western blot assay). **F**, **G** AGS cells were transfected with empty vector and GNB4-overexpression lentivirus. Nuclear and cytoplasmic protein extraction and western blotting of GNB4 and YAP1 were performed (**F**). Representative images showing immunofluorescence staining (scale bar: 20 μm) for YAP1 (green) (**G**). **H** Immunoprecipitation assay of AGS cells with and without GNB4 overexpression using an anti-YAP1 antibody. Immunoglobulin G (IgG) was used as a negative control. Western blotting was performed to determine YAP1, TEAD1/2/3/4, and GNB4 expression

GPCR signaling can activate or inhibit the Hippo–YAP1 signaling pathway through coupled G proteins [24]. In addition, several studies have suggested the important role of the Hippo pathway in the development of GC [35]. GSEA was conducted using the TCGA-STAD data to determine the correlation between GNB4 and Hippo pathway via CAMOIP online tool [27]. Consequently, high GNB4 expression signatures were identified reflecting the activation status of the Hippo signaling pathway (*P* < 0.05, Fig. 6A). Moreover, GNB4 expression showed a significant correlation with YAP1 (*R* = 0.38, *P* < 0.0001) but not TAZ (*R* = − 0.087, *P* = 0.079), two key effectors of the Hippo pathway, in TCGA database (Additional file 7: Fig. S7). Our data also showed that *H. pylori* infection obviously induced YAP1 protein expression in GC cell lines and clinical GC samples (Additional file 8: Fig. S8). Therefore, we investigated the effect of GNB4 on the activation of YAP1 and its downstream target genes such as CYR61 and CTGF. qRT-PCR analysis revealed that overexpression of GNB4 significantly enhanced YAP1, CYR61, and CTGF mRNA expression in GC cell lines (*P* < 0.01, Fig. 6B, C). As expected, similar results were obtained in western blot assays (Fig. 6D). Furthermore, following the treatment with increasing doses of VP, a YAP inhibitor; disrupting YAP1 interaction with TEADs; and promoting trypsin cleavage of YAP1 [36], we found no significant change in the expression of GNB4, suggesting an upstream regulation of YAP1 protein levels by GNB4 (Fig. 6E).

*H. pylori* promoted gastric carcinogenesis by inducing YAP1 nuclear translocation [21]. Next, we performed a nuclear/cytoplasmic protein extraction assay, and the results showed an increase in YAP1 nuclear accumulation following GNB4 overexpression, whereas cytoplasmic protein expression of YAP1 decreased in AGS cells (Fig. 6F). Immunofluorescence assay confirmed these results (Fig. 6G). Furthermore, we investigated the effect of GNB4 on the interaction between YAP1 and TEADs. The results of the co-IP assay revealed that overexpression of GNB4 enhanced the co-immunoprecipitation of YAP1 and TEAD1/2/3/4 in AGS cells (Fig. 6H). Overall, the above results show that GNB4 promotes the nuclear expression of YAP1 and its interaction with TEAD1/2/3/4, which in turn activates downstream target genes in GC cells.

### GNB4 induces GC malignancy through the Hippo–YAP1 pathway in vitro and in vivo

**Fig. 7:**
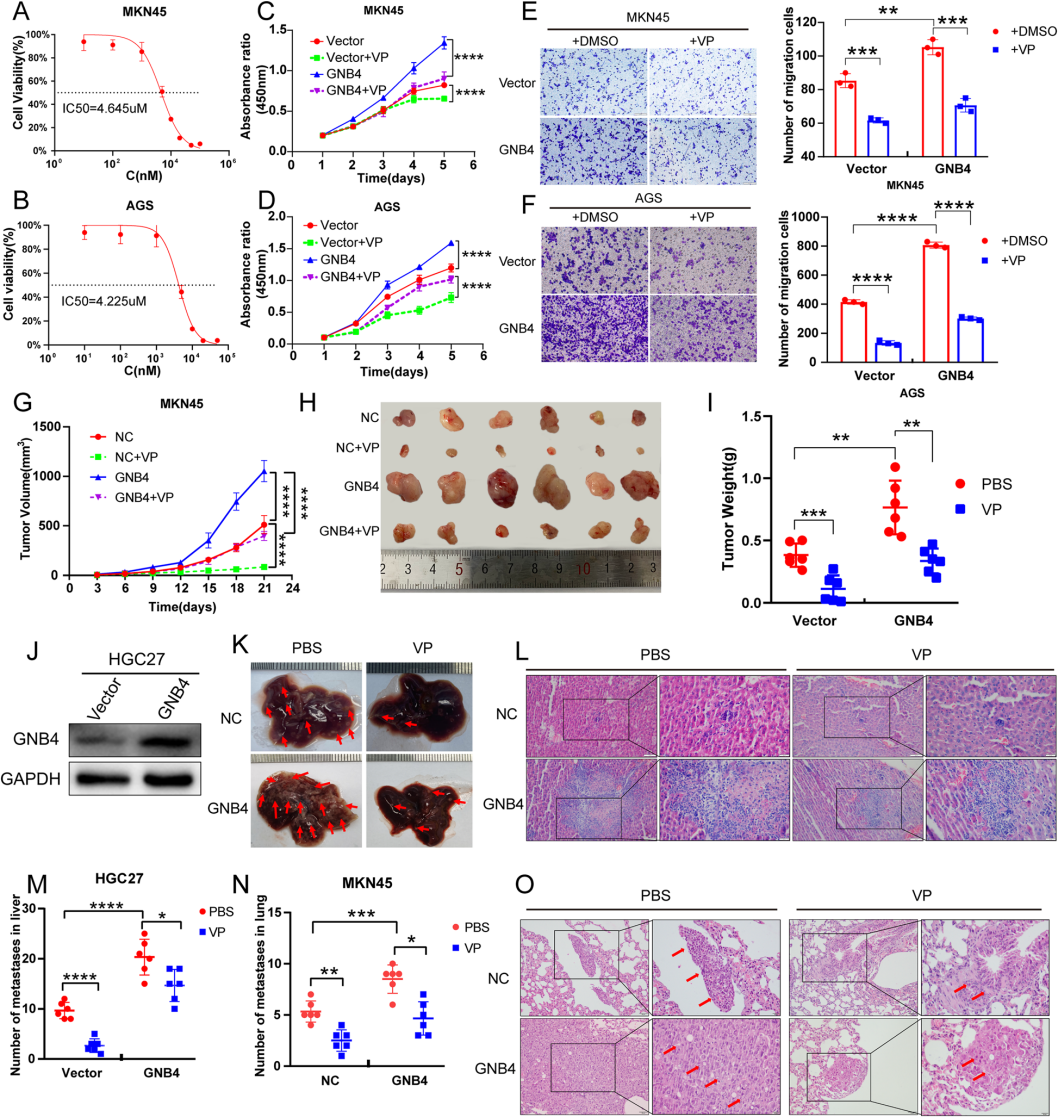
GNB4 induces GC malignancy through the Hippo–YAP1 pathway in vitro and in vivo. **A**, **B** MKN45 (**A**) and AGS (**B**) cells were treated with different concentrations of verteporfin (VP; YAP1 inhibitor), and half-maximal inhibitory concentrations (IC_50_) of the VP were measured by CCK-8 assay after 24 h. **C**–**F** VP was added to MKN45 and AGS cells with or without GNB4 overexpression for 24 h at concentrations obtained through IC_50_ analysis. CCK-8 (**C**, **D**) assay detected the proliferative capacity of cells, and transwell assays (**E**, **F**) monitored the migration ability of cells. Representative images (scale bar: 100 μm; left panels **E** and **F**) and histogram show the relative number (with reference to negative control) of cells that passed through the chamber membrane in each group (right panels **E** and **F**). **G**–**I** Subcutaneous tumor models (*n* = 6 mice per group) were established using stable GNB4-overexpressed MKN45 cells combined with VP intraperitoneal injection (100 mg/kg, once daily, 7 times). Growth curves (**G**) and tumor weights (**I**) were analyzed. Mass images of tumors collected from mice are shown (**H**). **J** Western blot analysis of GNB4 protein expression in HGC27 cells transfected with lentivirus vector or overexpressing exogenous GNB4. **K**–**O** Mouse xenograft assays: HGC27 and MKN45 cells with or without GNB4 overexpression were injected in the tail vein of athymic nude mice, and 3 weeks after cell inoculation, the mice were treated with VP (100 mg/kg) daily for 7 days (*n* = 6 mice per group). Representative mass images (scale bars: 1 mm, **K**) of mouse liver and representative H&E images (scale bar: 50 μm; inset: 20 μm) of liver samples (HGC27 cells injected, **L**) and lung samples (MKN45 cells injected, **O**) from the indicated groups of nude mice. The number of metastatic nodules in mouse liver (**M**) and lung (**N**) was analyzed

We further investigated whether GNB4 promotes gastric carcinogenesis via the Hippo–YAP1 pathway. For this purpose, we first determined the half maximal inhibitory concentration (IC_50_) of VP (YAP1 inhibitor) in MKN45 (IC_50_ = 4.645 nM) and AGS cells (IC_50_ = 4.225 nM) to assess their degree of tolerance to the drug (Fig. 7A, B). Next, based on the measured IC_50_ values, we performed CCK-8 and transwell assays to test whether VP could inhibit the pro-proliferative and migratory ability of GNB4 in GC cells. Our data indicated that treatment with VP significantly diminished the proliferation and migration, which was enhanced by GNB4 overexpression (*P* < 0.001, Fig. 7C–F). Taken together, GNB4 may have a cancer-promoting function through the Hippo–YAP1 pathway.

Next, we performed animal experiments to validate the data obtained with cell lines. We aimed to explore whether controlling GNB4 expression and YAP1 activity can be used as a potential antitumor therapeutic strategy for *H. pylori*-induced GC. Therefore, we subcutaneously injected GNB4-overexpressing or negative control MKN-45 cells into 6-week-old male BALB/c nude mice. These tumor-bearing mice were intraperitoneally injected with VP (100 mg/kg) or PBS once a week. The tumors were harvested on the 21st day and analyzed further. We found that GNB4 overexpression promoted tumor growth and increased tumor weight, but treatment with VP inhibited this effect (*P* < 0.01, Fig. 7G–I). Next, to clarify the effects of YAP1 on the metastasis of GC, we also established an HGC-27 cell line that stably overexpressed GNB4 (Fig. 7J). Then, GNB4-overexpressing and negative control HGC27 and MKN45 cells were injected into the tail vein of 6-week-old male BALB/c mice, followed by intraperitoneal injection of VP (100 mg/kg) or PBS once daily for 7 consecutive days in the third week. The mice were sacrificed 2 weeks after treatment, and their lungs and livers were collected. We observed that activation of GNB4 promoted the metastasis of GC with an increase in liver and pulmonary metastasis, and histopathological findings confirmed these results (Fig. 7K–O). However, the promotion of GC metastasis was diminished after treatment with VP (*P* < 0.05, Fig. 7K–O). Taken together, our findings revealed that inhibition of YAP1 suppressed GC malignancy induced by GNB4 overexpression.

## Discussion

The development of GC involves multiple genetic and epigenetic alterations, and the disease usually presents at an advanced stage. Previous studies have highlighted an important link between *H. pylori*-induced aberrant DNA methylation and gastric carcinogenesis [37, 38]. However, the mechanism of *H. pylori*-induced aberrant gene methylation in gastric carcinogenesis remains poorly understood. In this study, we demonstrated that *H. pylori* infection induced demethylation of the GNB4 promoter region and identified the differentially methylated CpG#5 site (chr3:179451746–179451745) in vitro and in vivo. Furthermore, TET1 contributed to GNB4 demethylation that led to the upregulation of GNB4 in GC. Furthermore, GNB4 promoted GC proliferation and metastasis via the Hippo**–**YAP1 pathway.

The accumulation of aberrant DNA methylation caused by *H. pylori* infection plays a crucial role in gastric carcinogenesis [4, 39, 40]. Consistent with previous studies [9, 41, 42], we found a high expression of GNB4 in GC tissues of humans and mice and its prognostic value in a public database and our cohort. In addition, we detected a significant increase in GNB4 expression in response to *H. pylori* infection in human and mouse gastric tissues*.* Furthermore, we identified the site (CpG#5) and promoter regions in the GNB4 gene, where significant demethylation occurs after *H. pylori* infection in GC cells and clinical GC samples of our cohort. Therefore, we speculate that *H. pylori* infection increases GNB4 expression by affecting demethylation modifications in its promoter region. Few studies have investigated the DNA methylation modulation of GNB4 in tumor tissues. For example, Wang et al. demonstrated that GNB4 was epigenetically silenced by DNA methylation in breast cancer cells [10], suggesting that GNB4 modulation varies in different tumor tissues.

Active demethylation is mediated by TET family proteins, which maintain the fidelity of DNA methylation patterns by mediating demethylation and altering the local chromatin environment for activating gene transcription [43–45]. Our data showed that *H. pylori* infection significantly enhanced the TET1 expression rather than DNMT1; this observation is consistent with a previous report that *H. pylori* infection did not induce the expression of DNMTs [46]. However, it is still controversial whether TET1, as a gene encoding a demethylase, plays an oncogenic or an oncogenic suppressor role in tumors. Duan et al. reported that TET1 played an inhibitory role in epithelial ovarian cancer by activating suppressors (DKK1 and SFRP2) of the Wnt/β-catenin signaling pathway [47]. Conversely, Zhao et al. found that *H. pylori* infection downregulated the expression of TET1 and inhibited the promoter methylation of KLF4, suggesting that TET1 is involved in the pathogenesis of *H. pylori* through another pathway [16]. We demonstrated that silencing of TET1 significantly suppressed GNB4 expression while increasing the methylation level of the GNB4 promoter region. Furthermore, activation of TET1 using DOX markedly increased the GNB4 expression. Moreover, the results of the ChIP assay confirmed the binding of TET1 on the GNB4 promoter region, supporting the role of TET1 in the demethylation regulation of GNB4. These data support that demethylation induced by *H. pylori* is TET1-dependent. The exact mechanism by which *H. pylori* infection regulates TET1 expression has not been investigated. Among bacterial factors of *H. pylori* associated with gastric carcinogenesis is the cytotoxin-associated gene A (CagA). When CagA interacts with integrins and transfers CagA and peptidoglycan peptides into cells through the bacterial hairs, the transcription factor NF-κB is activated, enters the nucleus, and induces the expression of pro-inflammatory cytokines and chemokines, especially IL-8 and CCL20 [48, 49]. It has been reported that CagA-induced IL-8 release through the Ras-Raf-Mek-Erk-NF-κB signaling pathway. Additionally, Acacia Lamb et al. reported that *H. pylori* stimulates NF-κB activation and expression of inflammatory cytokines through the CagA-dependent, TRAF6-mediated Lys 63-ubiquitination, and activation of TAK1 [50]. It can be seen that *H. pylori* activates NF-κB mainly through CagA, and NF-κB enters the nucleus and activates the expression of pro-inflammatory genes to induce an inflammatory response. Based on our results, we speculate that NF-κB regulates TET1 expression in response to *H. pylori* infection in GC cell lines. By investigating the upstream regulatory mechanisms of GNB4, we demonstrated that its expression was related to the demethylation induced by *H. pylori* in a TET1-dependent manner. The present study provides a novel mechanism of the upstream regulation of GNB4 expression.

The Hippo cascade is sensitive to environmental stimuli [51]. In particular, YAP1 is the key terminal effector of this pathway [51]. Several researchers have demonstrated that *H. pylori* promotes gastric tumorigenesis through the Hippo–YAP1 pathway [20, 21]. Therefore, the role of this pathway in the pro-cancer function of GNB4 should be further investigated. We observed that GNB4 overexpression significantly increased the expression of YAP1 and its target genes (CYR61 and CTGF), whereas inhibition of YAP1 expression with VP did not affect GNB4 expression. These results indicate that GNB4 regulates the YAP1 pathway (but not vice versa) in GC cells. TEADs, the nuclear DNA-binding proteins in the context of Hippo signaling, can be passively activated by YAP1 [52]. In our study, GNB4 overexpression promoted TEAD expression and nuclear accumulation of YAP1. The results of the co-IP assay showed that GNB4 overexpression enhanced the binding ability of YAP1 and TEADs. Our findings are similar to some previous studies, in which YAP1 promoted tumorigenesis by binding and activating TEADs in multiple tumors including GC [53–56]. In addition, functional experiments demonstrated that the cancer-promoting function of GNB4 was inhibited after the YAP1–TEADs interaction was disrupted by VP addition. Overall, these findings indicate that GNB4 facilitates the proliferation and metastasis of GC by regulating the Hippo–YAP1 pathway.

## Conclusions

**Fig. 8:**
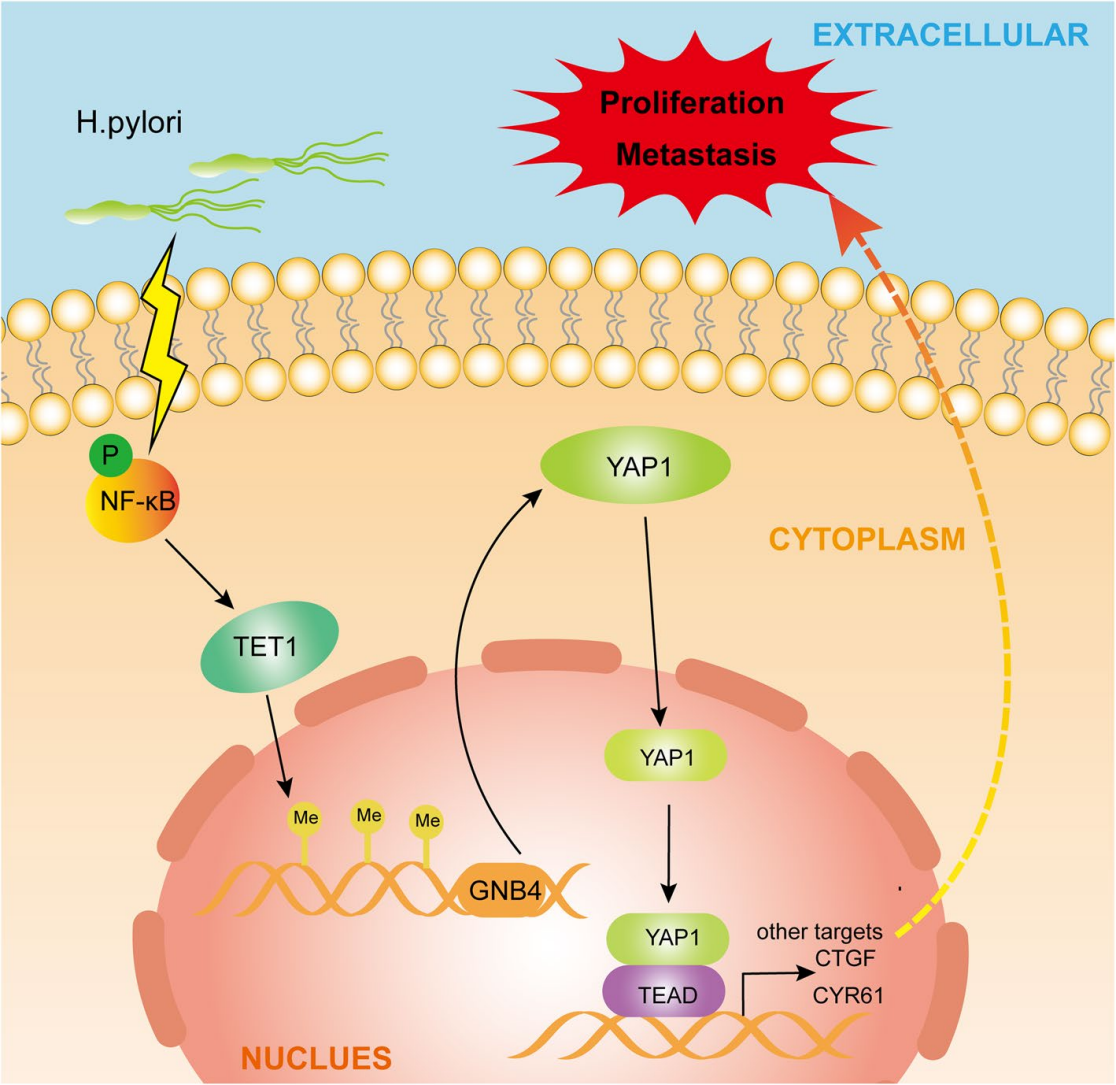
Schematic illustration of the molecular mechanism of GNB4 in *H. pylori*-induced GC. *H. pylori* induces GNB4 upregulation via the NF-κB-TET1 axis. TET1-mediated demethylation of GNB4 promoter leads to the transcriptional activation of GNB4, and high GNB4 expression contributes to GC progression via the Hippo–YAP1 pathway

We demonstrated the crucial role of GNB4 demethylation modification in *H. pylori*-induced GC and outlined the mechanism of the *H. pylori*-NF-κB-TET1-GNB4 demethylation-YAP1 pathway (Fig. 8). GNB4 overexpression is involved in the pathogenesis of *H. pylori* infection-induced GC. Therefore, GNB4 inhibitors can be explored as a novel therapeutic approach to improve clinical outcomes in patients with GC.

## Supplementary Information

**Additional file 1: Fig. S1.** GNB4 expression correlates with the GC clinical traits and is upregulated in GC cell lines. A. Area under the receiver operator characteristic (ROC) curve for GNB4 in the differential diagnosis of GC with and without lymph node metastasis (AUC = 0.576, P = 0.019). B, C. qRT-PCR (B) and western blot analysis (C) of GNB4 expression in GC cell lines. Data are presented as the mean ± SD of three independent experiments.

**Additional file 2: Fig. S2.** *H. pylori* infection induces GNB4 overexpression in vitro and in vivo. A. qRT-PCR analysis of GNB4 mRNA levels in MKN45, AGS, and GES1 cells infected with *H. pylori* 26695 or *H. pylori* SS1 for 6 h. B, C. AGS and GES1 cells were infected with *H. pylori* 26695 and *H. pylori* SS1 for 6 h. Quantification of mean fluorescence intensity for GNB4 positive staining in GES1 and AGS cells (B). Representative images of AGS cells (C) showing immunofluorescence staining (scale bar: 25 µm) for GNB4 (red), CagA (purple), and DAPI (blue). D. mRNA expression of GNB4 was significantly upregulated in *H. pylori*-infected gastric tissue (GSE13873).

**Additional file 3: Fig. S3.** Gene set enrichment analysis (GSEA) based on gene expression analysis of patients with GC (n = 433) in the GSE84437 dataset.

**Additional file 4: Fig. S4.** *H. pylori* triggers GC cells malignancy via regulating GNB4 expression. A-C. CCK-8 assay was performed to monitor the cell proliferation in AGS shControl or shGNB4 (A) and changes in proliferation of AGS shControl (B) and shGNB4 (C) uninfected or infected with *H. pylori* strains (26695 and SS1; 6 h). D, E. Live cell counts after 3,6,9 hours of *H. pylori* infection of MKN45 cells inoculated in 6-cm dishes and incubated for 72 hours. Representative images acquired by the Countstar camera at different time points after *H. pylori* strains infection(D) and quantitative analysis of live cells (E). F, G. EdU assays were conducted in AGS shControl and shGNB4 uninfected or infected with *H. pylori* strains (26695 and SS1; 6 h) to compare the percentage of cells in S phase (scale bar: 100 μm). DAPI staining detected total cells, whereas EdU staining identified cells with active DNA replication. Representative images (F) and quantification data (G) are shown. H, I. Wound healing assays were performed to compare the migration capabilities of AGS shControl and shGNB4 uninfected or infected with *H. pylori* strains (26695 and SS1; 6 h). The difference in cell margin at 0 and 48 h showed the moving track of cells (scale bar: 200 μm) (H), and the percentage of the healed area was quantified (I). J-L. Transwell assays of AGS shControl and shGNB4 uninfected or infected with *H. pylori* strains (26695 and SS1; 6 h) were performed to measure their migration and invasion abilities (scale bar: 100 μm). Representative images (J) and bar charts show the number of cells which passed through the chamber membrane in each group (K, L). Data are presented as the mean ± SD of three independent experiments.

**Additional file 5: Fig. S5.** Methylation level of four CpG sites of the GNB4 promoter region in MKN45 cells uninfected or infected with *H. pylori* strains (26695 and SS1) for 6 h.

**Additional file 6: Fig. S6.** *H. pylori* promotes TET1 expression by regulating NF-κB in GC cell lines. A. Correlation analysis between GNB4 expression and DNA methylation transferase genes (DNMT1, DNMT3A, and DNMT3B), TET2, and TET3 in patients with GC via GEPIA database (Spearman method, *P* > 0.05). B. Western blot analysis of GNB4 and TET1 protein expression in MKN45 cells treated with GNB4 shRNA.

**Additional file 7: Fig. S7.** Correlation analysis between the expression of GNB4 and Hippo pathway effectors (YAP1 and TAZ) using the GEPIA database (Spearman method).

**Additional file 8: Fig. S8.** *H. pylori* activates YAP1 expression in vitro and in clinical samples. A. Western blot analysis of CagA, YAP1, and GNB4 expression in AGS and MKN45 cells uninfected or infected with *H. pylori* strains (26695 and SS1; 6 h). B. Western blot analysis of YAP1 expression in *H. pylori*-positive (HP+) and *H. pylori*-negative (HP-) GC tumor samples.

**Additional file 9: Table S1.** Target sequences of siRNA and shRNA used in this study; **Table S2.** PCR primer sequences. **Table S3.** Antibodies used in western blotting; **Table S4.** Sequences of primers used in MSP, pyrosequencing, and mass spectrometry methylation detection. **Table S5.** ChIP-qPCR primer sequences.

**Additional file 10: Fig. S9.** Uncropped western blots related to results.

**Additional file 11: Fig. S10.** Raw figure of MSP.

## Data Availability

The datasets generated during and/or analyzed during the current study are available from the corresponding author upon reasonable request.

## Abbreviations

GC: Gastric cancer
*H. pylori*: *Helicobacter pylori*
GNB4: Guanine nucleotide-binding protein subunit beta-4
TET: Ten–eleven translocation
5mC: 5-Methylcytosine
5hmC: 5-Hydroxymethylcytosine
YAP1: Yes-associated protein1
TAZ: Transcriptional co-activator with PDZ-binding motif
CYR61: Cysteine-rich angiogenic inducer 61
CTGF: Connective tissue growth factor
DNMTs: DNA methylation transferases
GEO: Gene expression omnibus
TCGA: The Cancer Genome Atlas
ROC: Receiver operator characteristic
AUC: Area under the ROC curve
EMT: Epithelial-mesenchymal transition
IC50: Half maximal inhibitory concentration
qRT-PCR: Real-time quantitative polymerase chain reaction
MSP: Methylation-specific PCR
DMR: Differentially methylated region
DOX: Doxycycline
VP: Verteporfin
PBS: Phosphate-buffered saline
MNU: *N*-methyl-*N*-nitrosourea
ChIP: Chromatin immunoprecipitation
Co-IP: Co-immunoprecipitation
GSEA: Gene set enrichment analysis
IHC: Immunohistochemistry
H&E: Hematoxylin-eosin
