# Supplementary figures and images for: Helicobacter pylori-induced aberrant demethylation and expression of GNB4 promotes gastric carcinogenesis via the Hippo–YAP1 pathway
Source: BMC Med. 2023 Apr 5;21:134. doi: 10.1186/s12916-023-02842-6 (PMC10073623; doi:10.1186/s12916-023-02842-6)

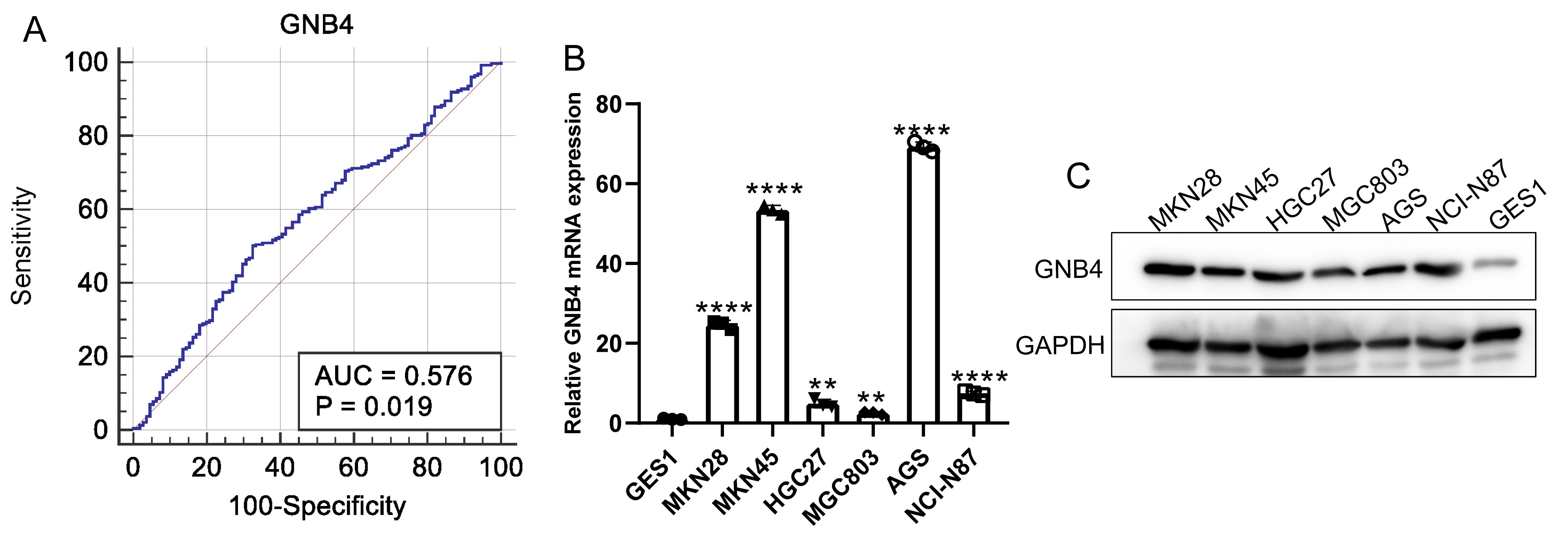

Supplement: Supplementary file 1 — Additional file 1: Fig. S1. GNB4 expression correlates with the GC clinical traits and is upregulated in GC cell lines. A. Area under the receiver operator characteristic (ROC) curve for GNB4 in the differential diagnosis of GC with and without lymph node metastasis (AUC = 0.576, P = 0.019). B, C. qRT-PCR (B) and western blot analysis (C) of GNB4 expression in GC cell lines. Data are presented as the mean ± SD of three independent experiments. [file 12916_2023_2842_MOESM1_ESM.tif]

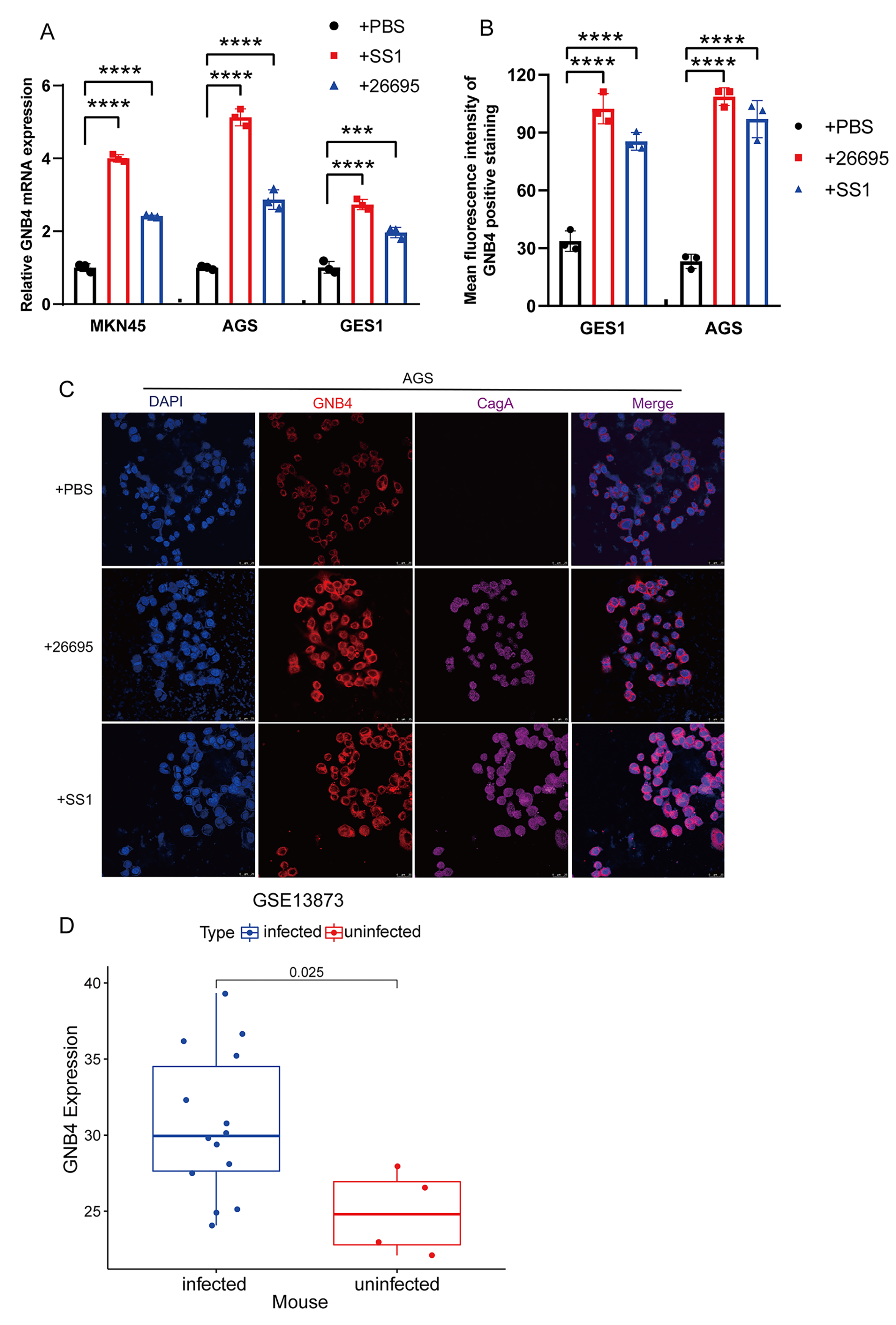

Supplement: Supplementary file 2 — Additional file 2: Fig. S2. H. pylori infection induces GNB4 overexpression in vitro and in vivo. A. qRT-PCR analysis of GNB4 mRNA levels in MKN45, AGS, and GES1 cells infected with H. pylori 26695 or H. pylori SS1 for 6 h. B, C. AGS and GES1 cells were infected with H. pylori 26695 and H. pylori SS1 for 6 h. Quantification of mean fluorescence intensity for GNB4 positive staining in GES1 and AGS cells (B). Representative images of AGS cells (C) showing immunofluorescence staining (scale bar: 25 µm) for GNB4 (red), CagA (purple), and DAPI (blue). D. mRNA expression of GNB4 was significantly upregulated in H. pylori-infected gastric tissue (GSE13873). [file 12916_2023_2842_MOESM2_ESM.tif]

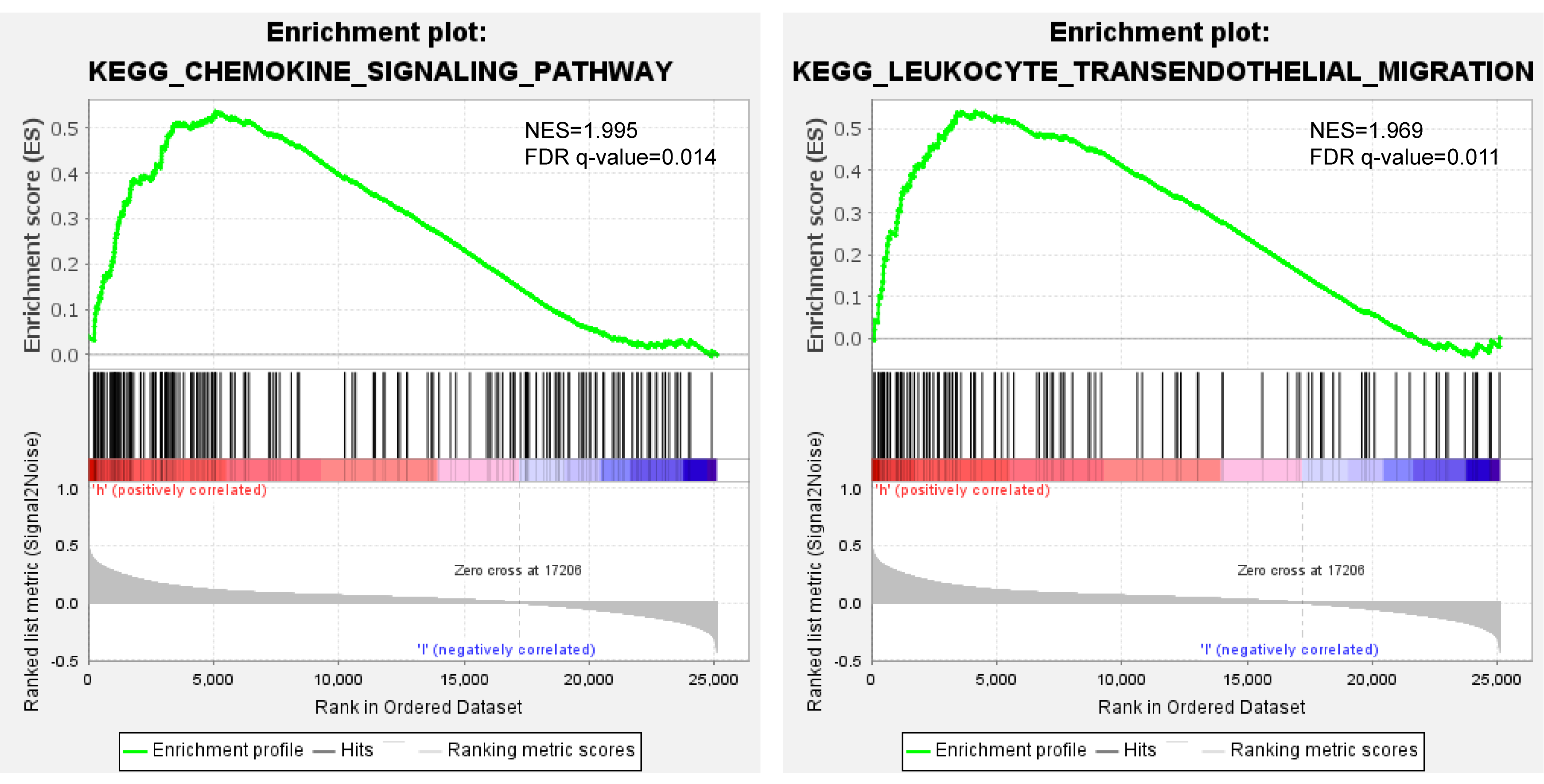

Supplement: Supplementary file 3 — Additional file 3: Fig. S3. Gene set enrichment analysis (GSEA) based on gene expression analysis of patients with GC (n = 433) in the GSE84437 dataset. [file 12916_2023_2842_MOESM3_ESM.tif]

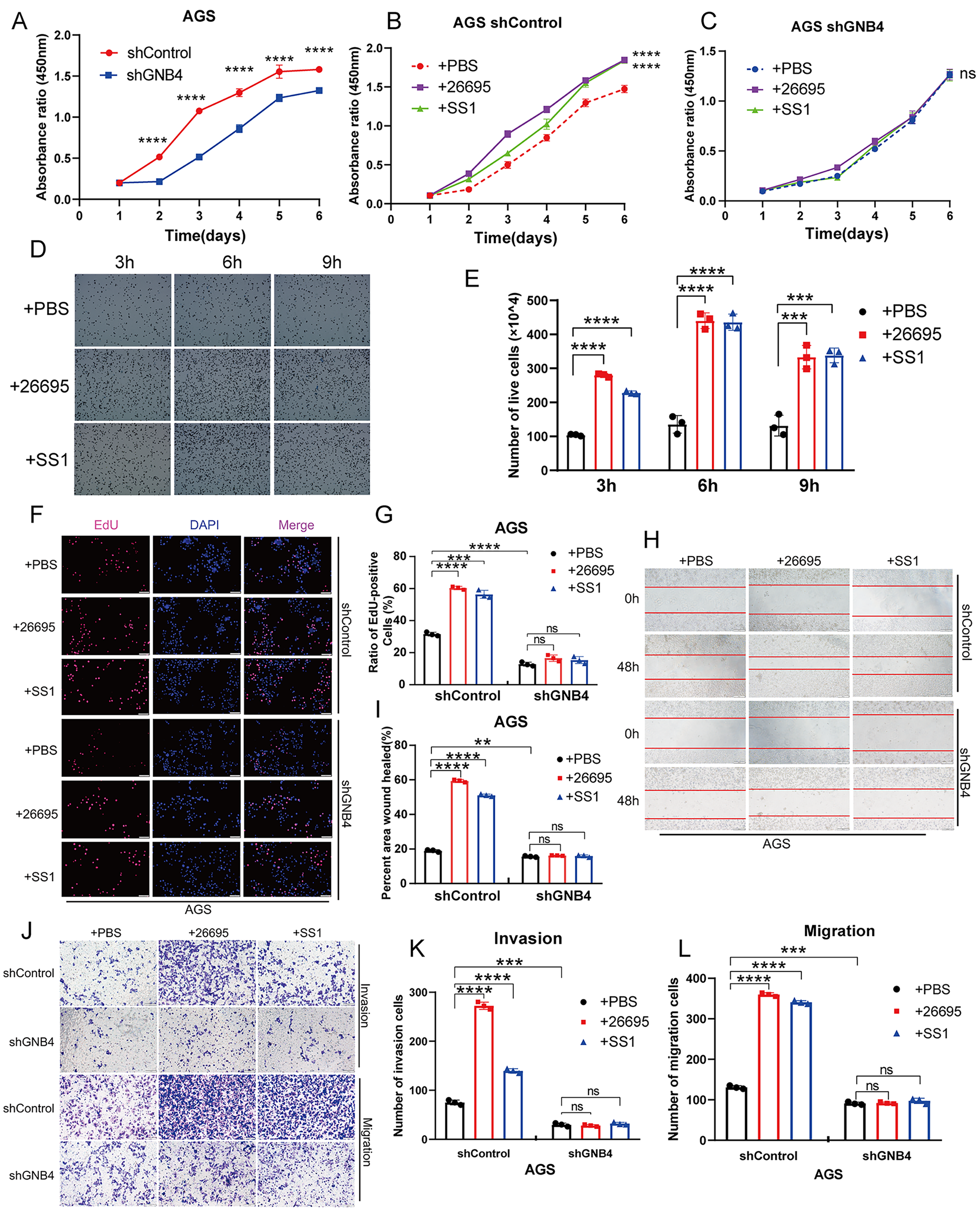

Supplement: Supplementary file 4 — Additional file 4: Fig. S4. H. pylori triggers GC cells malignancy via regulating GNB4 expression. A-C. CCK-8 assay was performed to monitor the cell proliferation in AGS shControl or shGNB4 (A) and changes in proliferation of AGS shControl (B) and shGNB4 (C) uninfected or infected with H. pylori strains (26695 and SS1; 6 h). D, E. Live cell counts after 3,6,9 hours of H. pylori infection of MKN45 cells inoculated in 6-cm dishes and incubated for 72 hours. Representative images acquired by the Countstar camera at different time points after H. pylori strains infection(D) and quantitative analysis of live cells (E). F, G. EdU assays were conducted in AGS shControl and shGNB4 uninfected or infected with H. pylori strains (26695 and SS1; 6 h) to compare the percentage of cells in S phase (scale bar: 100 μm). DAPI staining detected total cells, whereas EdU staining identified cells with active DNA replication. Representative images (F) and quantification data (G) are shown. H, I. Wound healing assays were performed to compare the migration capabilities of AGS shControl and shGNB4 uninfected or infected with H. pylori strains (26695 and SS1; 6 h). The difference in cell margin at 0 and 48 h showed the moving track of cells (scale bar: 200 μm) (H), and the percentage of the healed area was quantified (I). J-L. Transwell assays of AGS shControl and shGNB4 uninfected or infected with H. pylori strains (26695 and SS1; 6 h) were performed to measure their migration and invasion abilities (scale bar: 100 μm). Representative images (J) and bar charts show the number of cells which passed through the chamber membrane in each group (K, L). Data are presented as the mean ± SD of three independent experiments. [file 12916_2023_2842_MOESM4_ESM.tif]

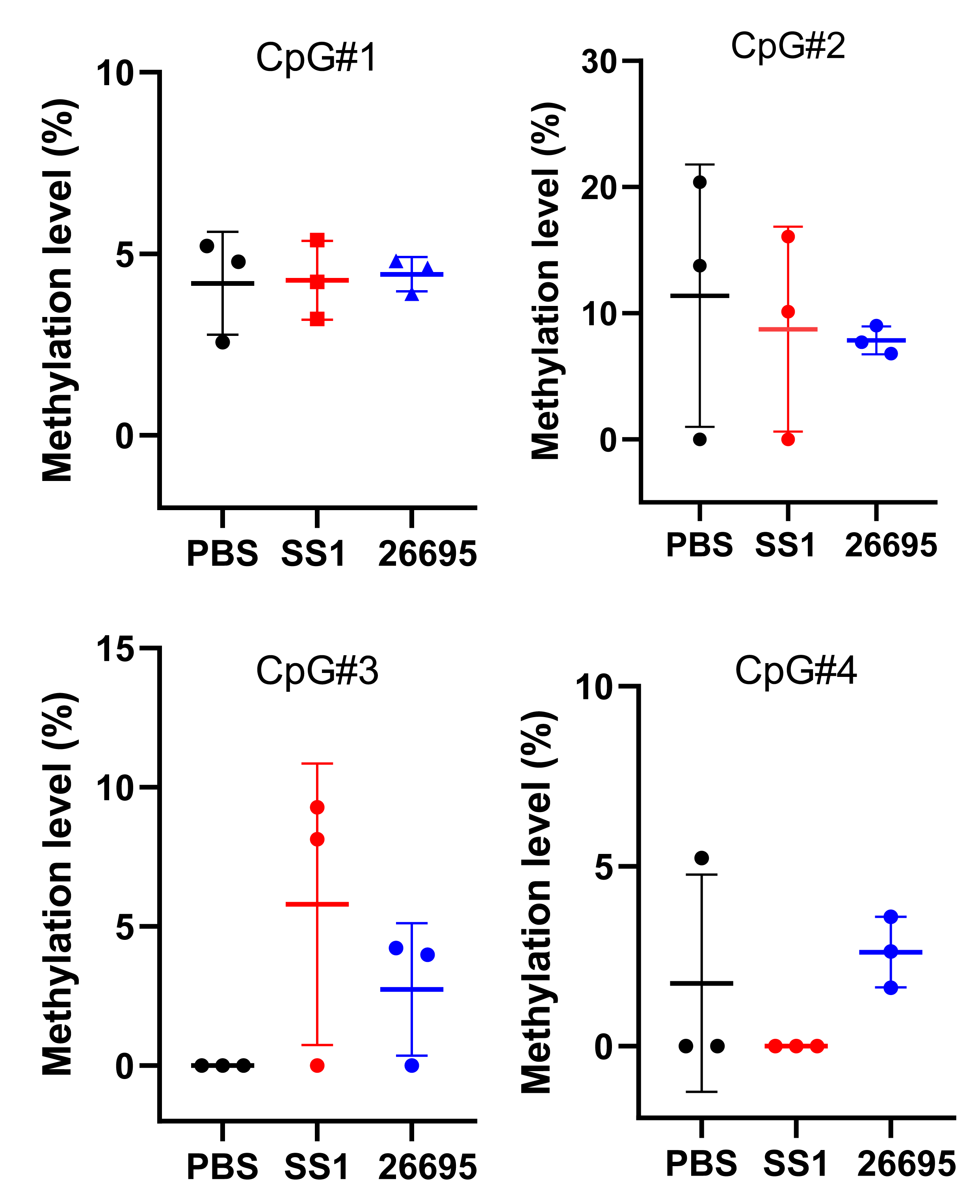

Supplement: Supplementary file 5 — Additional file 5: Fig. S5. Methylation level of four CpG sites of the GNB4 promoter region in MKN45 cells uninfected or infected with H. pylori strains (26695 and SS1) for 6 h. [file 12916_2023_2842_MOESM5_ESM.tif]

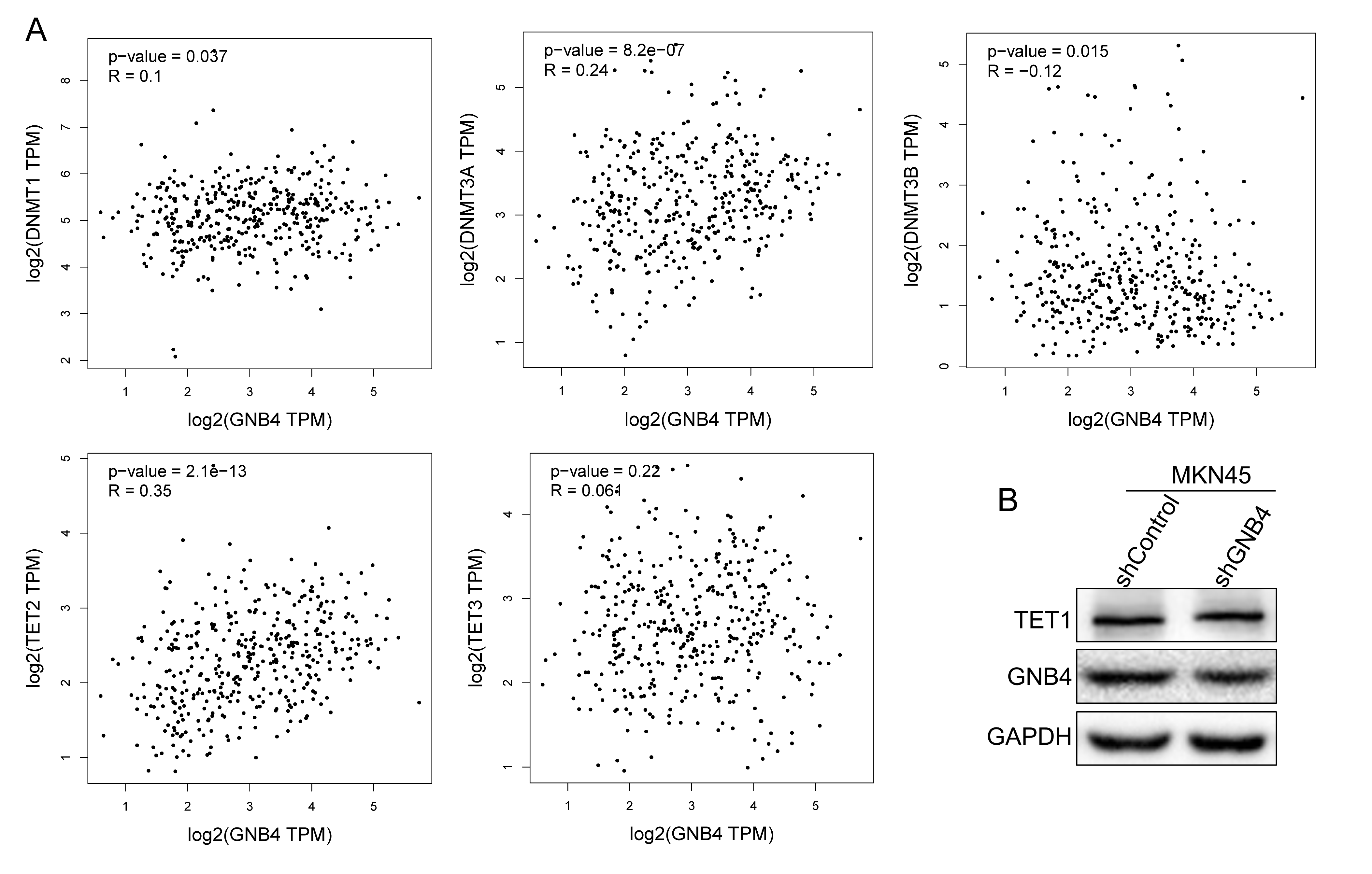

Supplement: Supplementary file 6 — Additional file 6: Fig. S6. H. pylori promotes TET1 expression by regulating NF-κB in GC cell lines. A. Correlation analysis between GNB4 expression and DNA methylation transferase genes (DNMT1, DNMT3A, and DNMT3B), TET2, and TET3 in patients with GC via GEPIA database (Spearman method, P > 0.05). B. Western blot analysis of GNB4 and TET1 protein expression in MKN45 cells treated with GNB4 shRNA. [file 12916_2023_2842_MOESM6_ESM.tif]

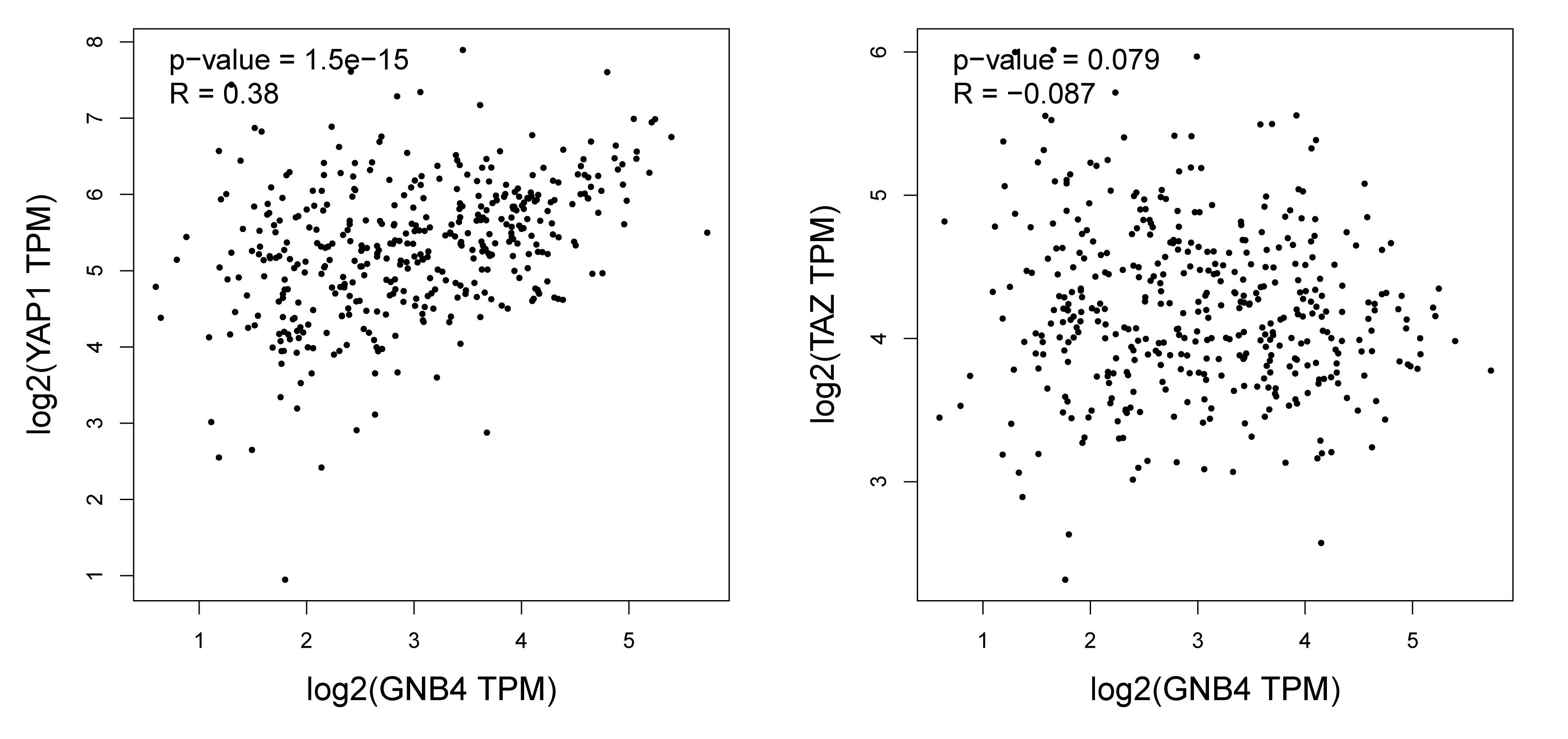

Supplement: Supplementary file 7 — Additional file 7: Fig. S7. Correlation analysis between the expression of GNB4 and Hippo pathway effectors (YAP1 and TAZ) using the GEPIA database (Spearman method). [file 12916_2023_2842_MOESM7_ESM.tif]

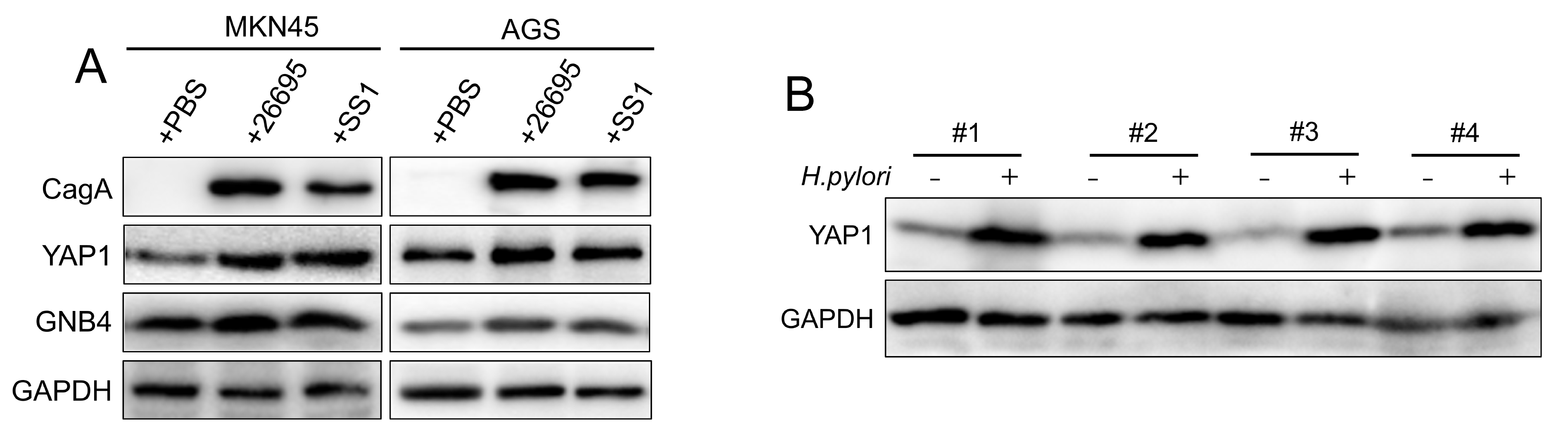

Supplement: Supplementary file 8 — Additional file 8: Fig. S8. H. pylori activates YAP1 expression in vitro and in clinical samples. A. Western blot analysis of CagA, YAP1, and GNB4 expression in AGS and MKN45 cells uninfected or infected with H. pylori strains (26695 and SS1; 6 h). B. Western blot analysis of YAP1 expression in H. pylori-positive (HP+) and H. pylori-negative (HP-) GC tumor samples. [file 12916_2023_2842_MOESM8_ESM.tif]

Figure 4C

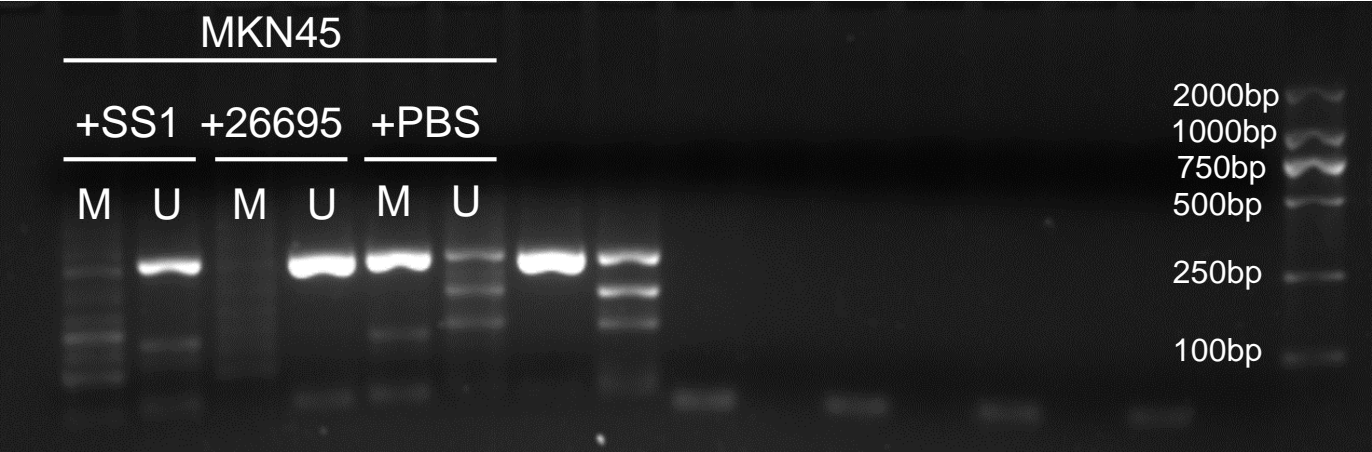

Figure 4E

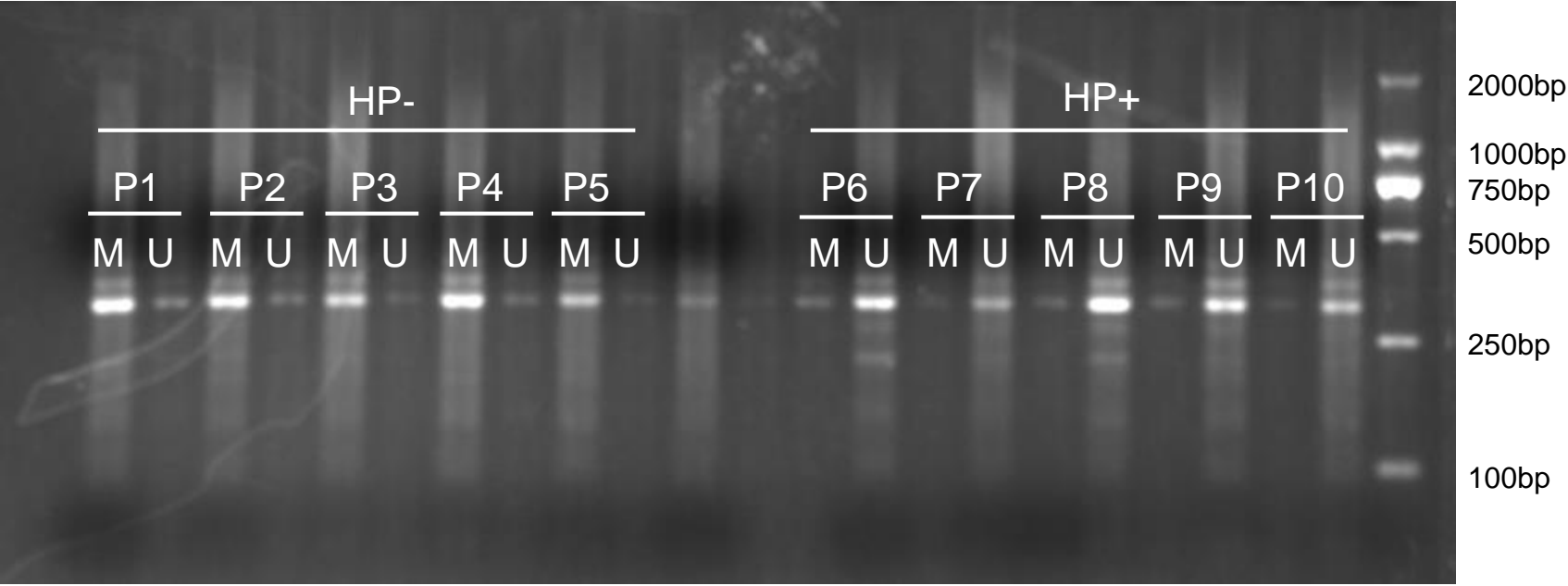

Figure 5E

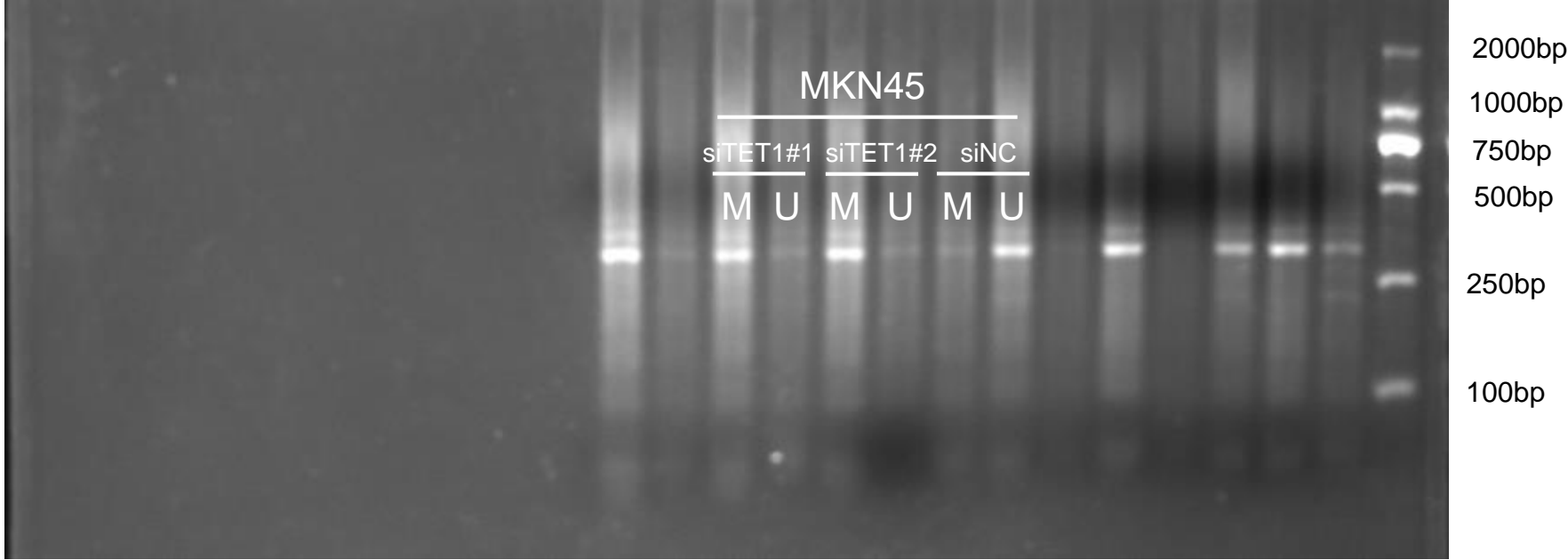

Supplement: Supplementary file 11 — Additional file 11: Fig. S10. Raw figure of MSP. [file 12916_2023_2842_MOESM11_ESM.pdf]
