# Supplementary material for: Helicobacter pylori-induced aberrant demethylation and expression of GNB4 promotes gastric carcinogenesis via the Hippo–YAP1 pathway
Source: BMC Med. 2023 Apr 5;21:134. doi: 10.1186/s12916-023-02842-6 (PMC10073623; doi:10.1186/s12916-023-02842-6)
Supplement: Supplementary file 9 — Additional file 9: Table S1. Target sequences of siRNA and shRNA used in this study; Table S2. PCR primer sequences. Table S3. Antibodies used in western blotting; Table S4. Sequences of primers used in MSP, pyrosequencing, and mass spectrometry methylation detection. Table S5. ChIP-qPCR primer sequences. [file 12916_2023_2842_MOESM9_ESM.docx]

**Table S1. Target sequences of siRNA, shRNA used in this study.**

| Gene symbol | Target sequences (5’→3’) |
| --- | --- |
| siTET1#1 | GAGAATAGGTATGGTCAAA |
| siTET1#2 | CTGTCTTGATCGAGTTATA |
| shGNB4 | GCTCGGAAAGCATGTAATGAT |

**Table S2. PCR primers sequences**

| Name | Primer sequences (5' → 3') |
| --- | --- |
| GNB4 (Forward) | TGGAACAGTTGAGGCAAGAAG |
| GNB4 (Reverse) | TGAACAAGCGTTGCATCATTACA |
| GNB4-m (Forward) | CAGGAGGCTGAACAGCTTCG |
| GNB4-m (Reverse) | GGCCCACGGAGTCCATATTA |
| TET1 (Forward) | CGCTACGAAGCACCTCTCTTA |
| TET1(Reverse) | CTTGCATTGGAACCGAATCATTT |
| TET1 -m (Forward) | ACACAGTGGTGCTAATGCAG |
| TET1-m (Reverse) | AGCATGAACGGGAGAATCGG |
| TET2 (Forward) | ATACCCTGTATGAAGGGAAGCC |
| TET2(Reverse) | CTTACCCCGAAGTTACGTCTTTC |
| TET3 (Forward) | TCCAGCAACTCCTAGAACTGAG |
| TET3(Reverse) | AGGCCGCTTGAATACTGACTG |
| YAP1(Forward) | TAGCCCTGCGTAGCCAGTTA |
| YAP1(Reverse) | TCATGCTTAGTCCACTGTCTGT |
| TAZ(Forward) | CACCGTGTCCAATCACCAGTC |
| TAZ(Reverse) | TCCAACGCATCAACTTCAGGT |
| CYR61(Forward) | GGTCAAAGTTACCGGGCAGT |
| CYR61(Reverse) | GGAGGCATCGAATCCCAGC |
| CTGF(Forward) | CAGCATGGACGTTCGTCTG |
| CTGF(Reverse) | AACCACGGTTTGGTCCTTGG |

m: mouse; the rest of the primers are human.

**Table S3. Antibodies utilized in Western Blot.**

| Antibody | Dilution ratio | Source |
| --- | --- | --- |
| Anti-GNB4 | 1:2500 | Proteintech Group, Chicago, IL, USA |
| Anti-TET1 | 1:1000 | ABclonal, Wuhan, Hubei, China |
| Anti-GAPDH | 1:5000 | Proteintech Group, Chicago, IL, USA |
| Anti-Survivin | 1:1000 | Proteintech Group, Chicago, IL, USA |
| Anti-Vimentin | 1:1000 | Proteintech Group, Chicago, IL, USA |
| Anti-N-cadherin | 1:1000 | ABclonal, Wuhan, Hubei, China |
| Anti-E-cadherin | 1:1000 | BD, Franklin Lakes, NJ, USA |
| Anti-CagA | 1:1000 | GeneTex, San Antonio, TX, USA |
| YAP1 | 1:1000 | CST, Boston, MA, USA |
| TEAD1/2/3/4 | 1:1000 | ABclonal, Wuhan, Hubei, China |
| CTGF | 1:1000 | ABclonal, Wuhan, Hubei, China |
| CYR61 | 1:1000 | ABclonal, Wuhan, Hubei, China |

**Table S4. Primers sequences of MSP, pyrosequencing and mass spectrometry methylation detection (MSMD).**

| Experimental name | Gene name | Primer sequences (5' → 3') |
| --- | --- | --- |
| MSP | GNB4-U(Forward) | GGAAAGGAAATTGGTTATGTTATTTAG |
| MSP | GNB4-U(Reverse) | CACCTCCCAACAACCAACTCC |
| MSP | GNB4-M(Forward) | GGAAAGGAAATTGGTTACGTTATTTAG |
| MSP | GNB4-M(Reverse) | CACCTCCCAACAACCAACTCC |
| Pyrosequencing | GNB4-1(Forward) | GTTTTTTGTTAGAAGGAGAAGAAAAATA |
| Pyrosequencing | GNB4-1(Reverse) | AAAAACCTAAATAAAACCTTTAAAATCTTC |
| Pyrosequencing | GNB4-2(Forward) | GTTTTTTGTTAGAAGGAGAAGAAAAATA |
| Pyrosequencing | GNB4-2(Reverse) | CCTAAATAAAACCTTTAAAATCTTCTAACT |
| MSMD | GNB4(Forward) | GTTTATTTGGAGGGAGTTTAGGTT |
| MSMD | GNB4(Reverse) | ATTTTTCTTCTCCTTCTAACAAAAAAC |

MSP：Methylation-specific PCR

**Table S5. ChIP-qPCR primers sequences**

| Name | Primer sequences (5' → 3') |
| --- | --- |
| Site1(Forward) | CACTTAGACGGGCGCTACTTA |
| Site1(Reverse) | TTCATTCCCGGGCAGTTTCG |
| Site2(Forward) | CCACGCCACTTAGACGGG |
| Site2(Reverse) | TCCCGGGCAGTTTCGTTTTC |
