# Supplementary material for: Helicobacter pylori-induced aberrant demethylation and expression of GNB4 promotes gastric carcinogenesis via the Hippo–YAP1 pathway
Source: BMC Med. 2023 Apr 5;21:134. doi: 10.1186/s12916-023-02842-6 (PMC10073623; doi:10.1186/s12916-023-02842-6)
Supplement: Supplementary file 10 — Additional file 10: Fig. S9. Uncropped western blots related to results. [file 12916_2023_2842_MOESM10_ESM.pdf]

WB original image

Figure S1C

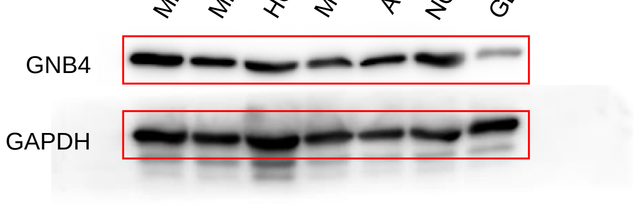

Figure 1G

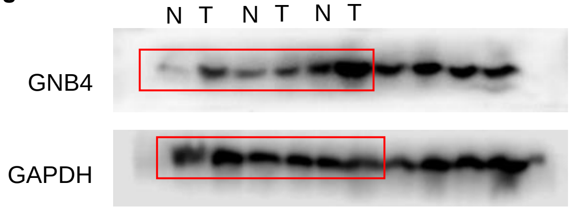

Figure 2B

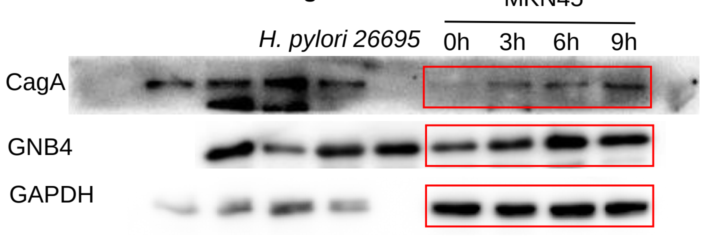

Figure 1G

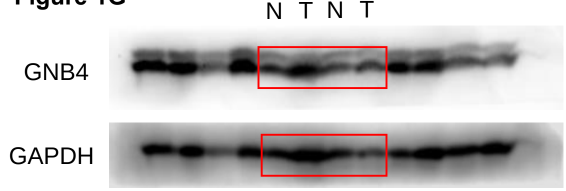

Figure 2A

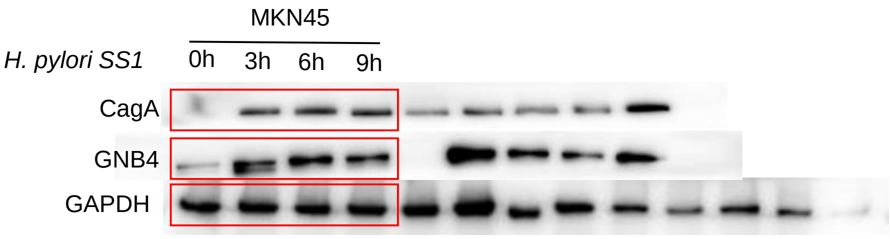

Figure 2A

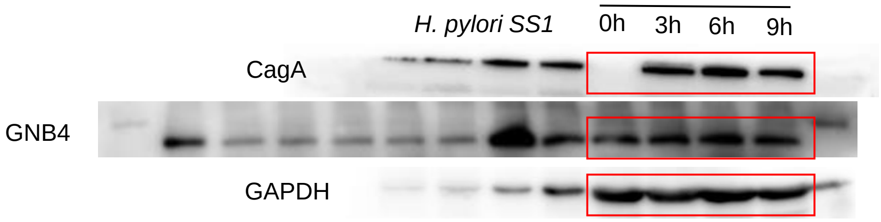

Figure 2B

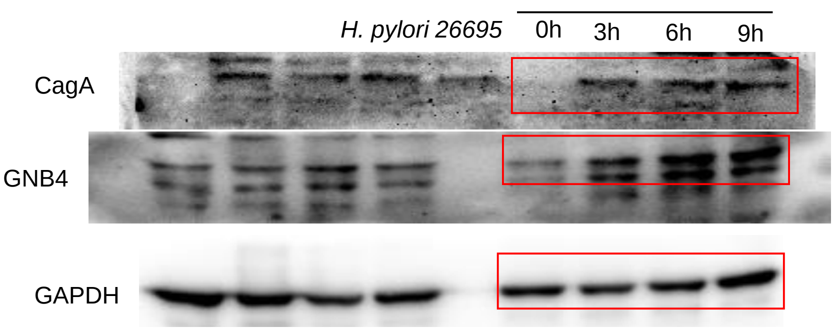

Figure 2C

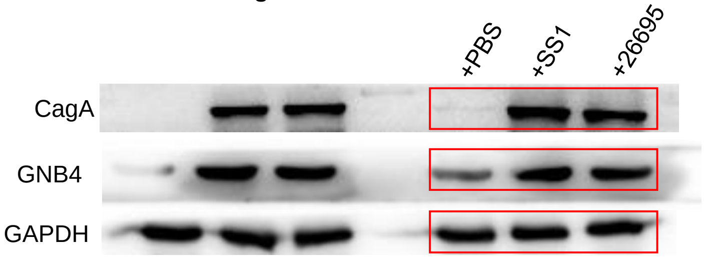

Figure 3O

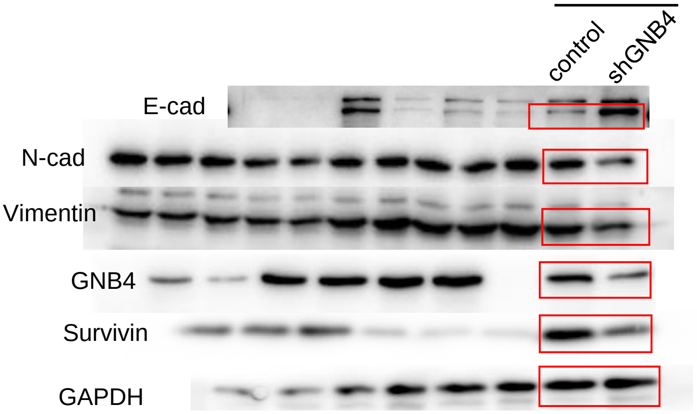

Figure 3O

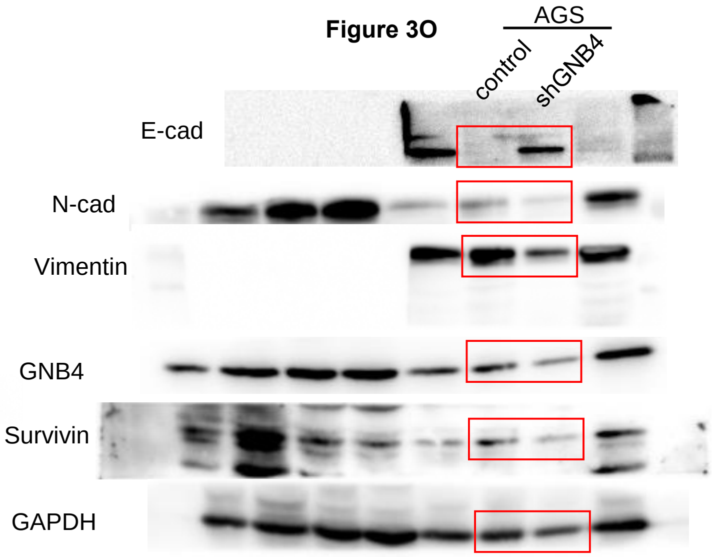

Figure 4D

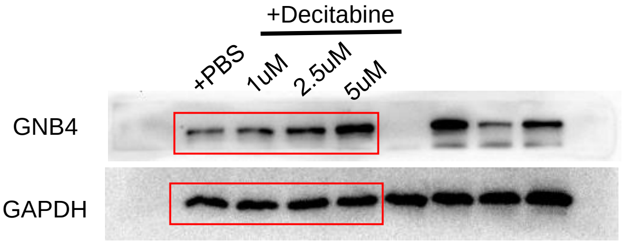

Figure 5C

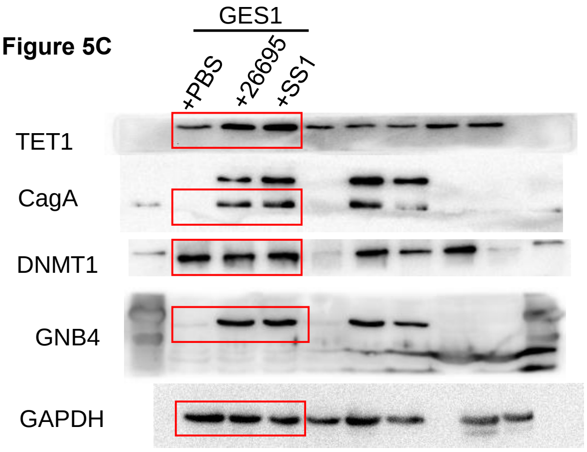

Figure5C

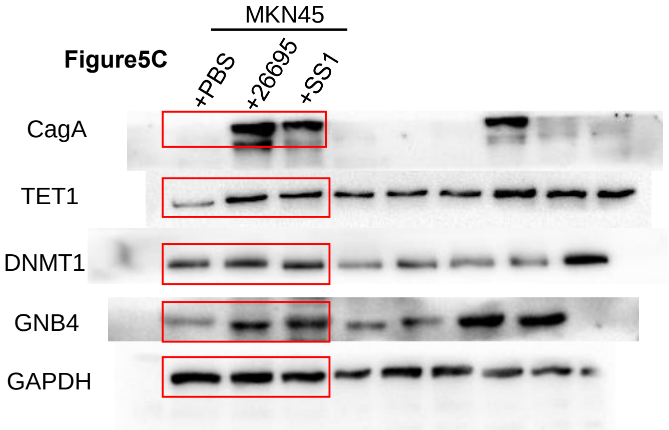

Figure5D

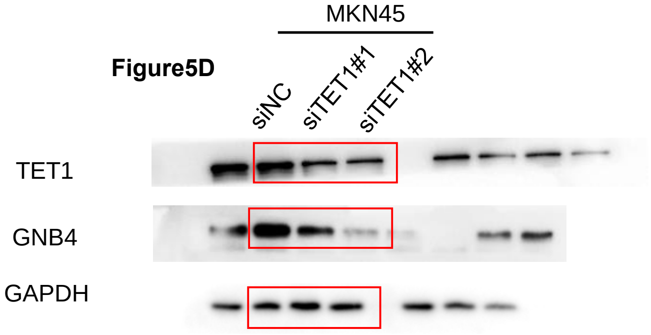

Figure S6B

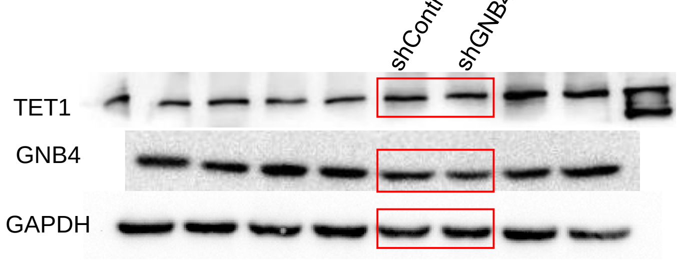

Figure 5F

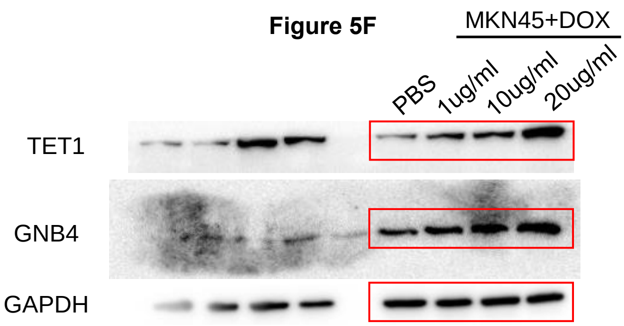

Figure6D

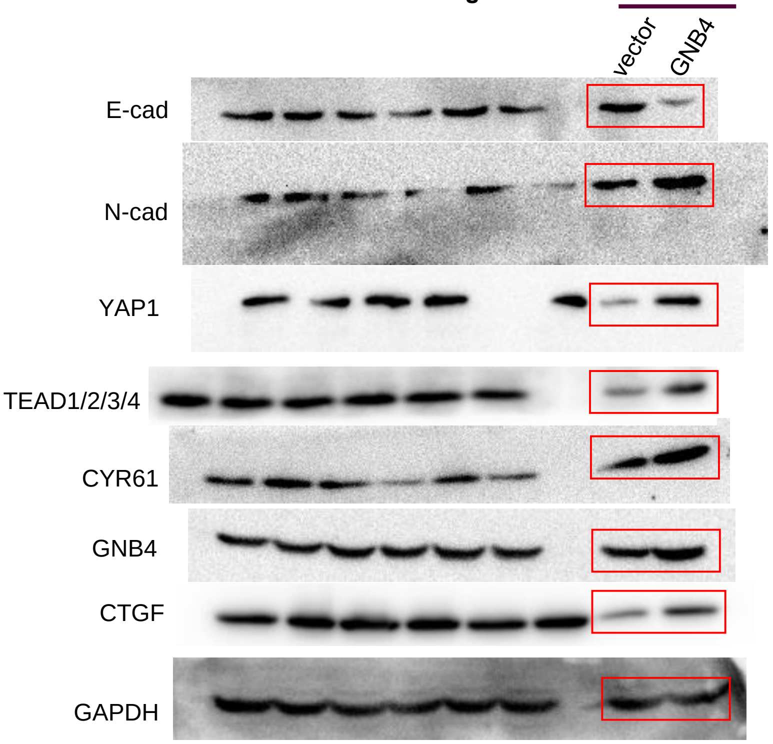

Figure 6D

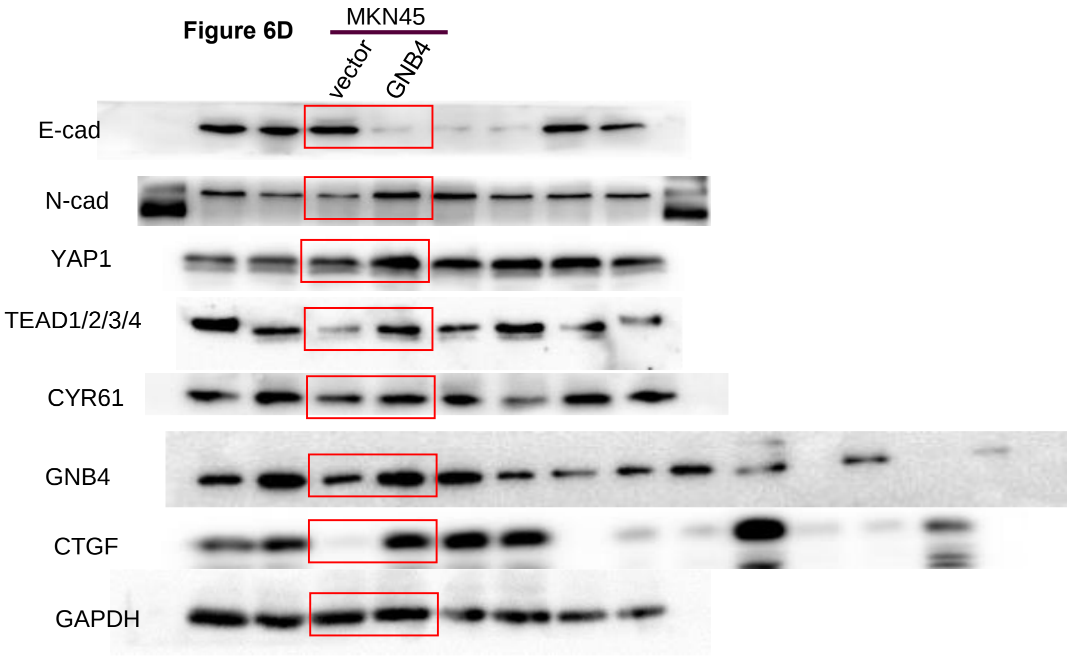

Figure 6E

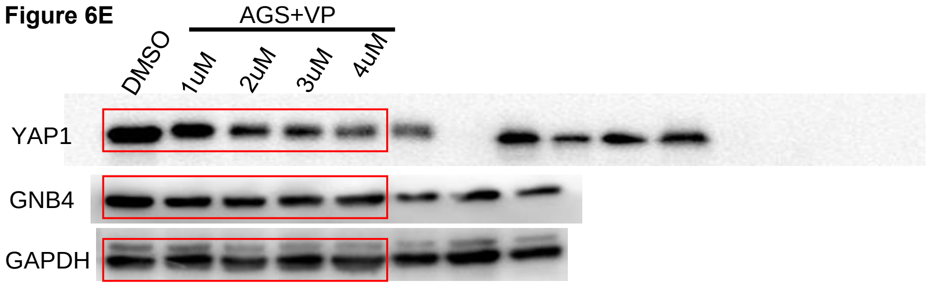

Figure 6E

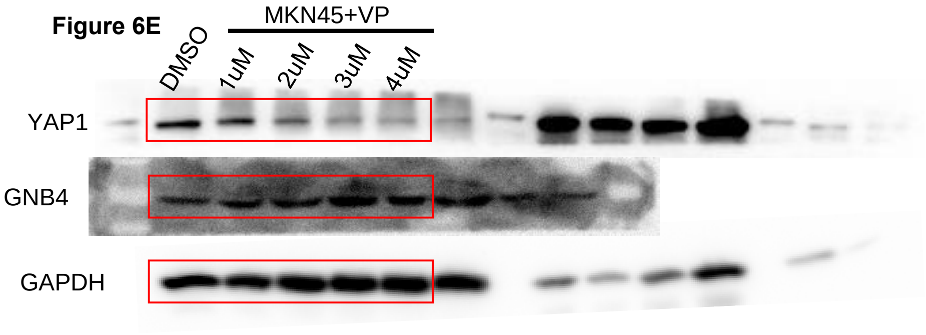

Figure 6F

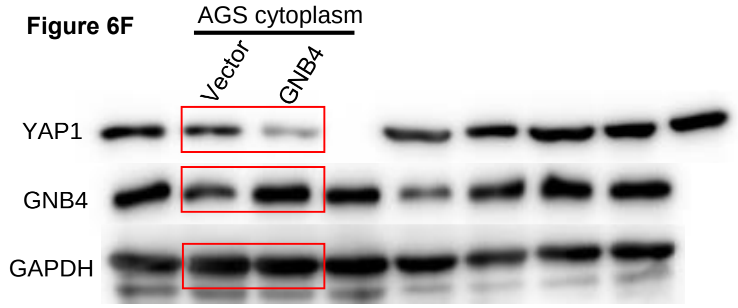

Figure 6F

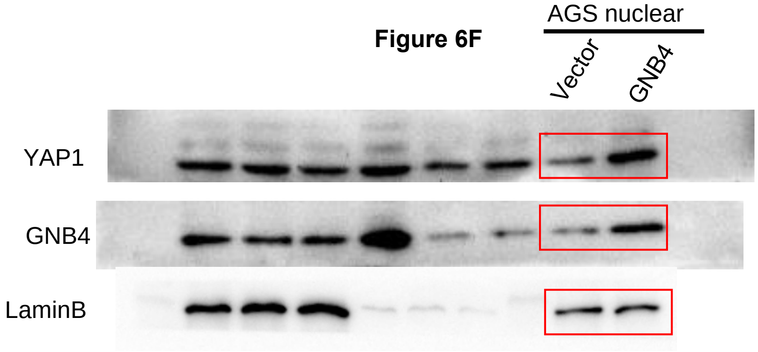

Figure 6H

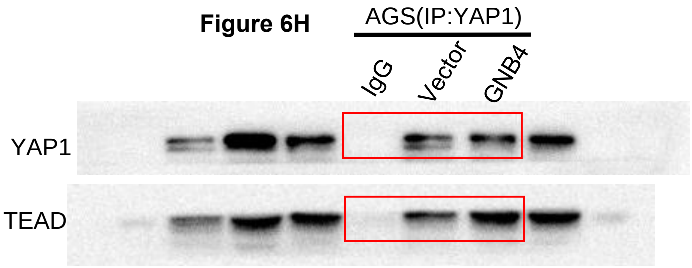

Figure6H

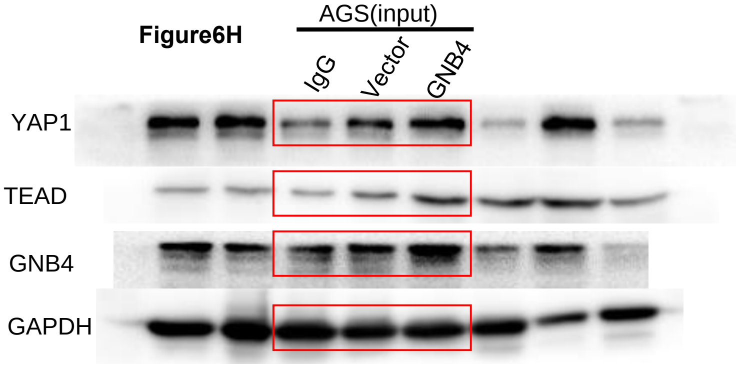

Figure 7J

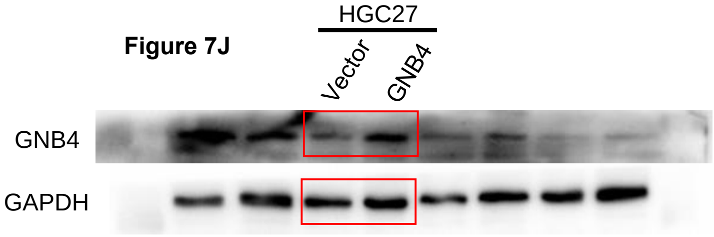

Figure S8A

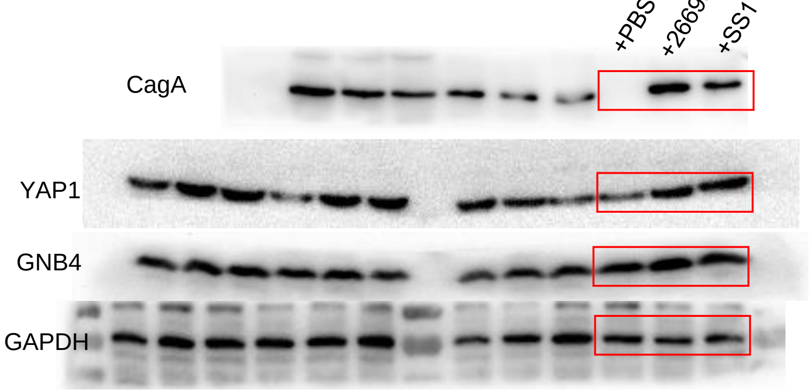

Figure S8A

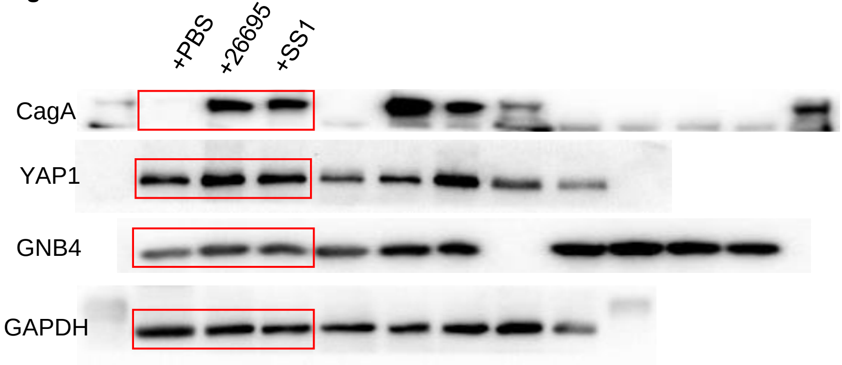

Figure S8B

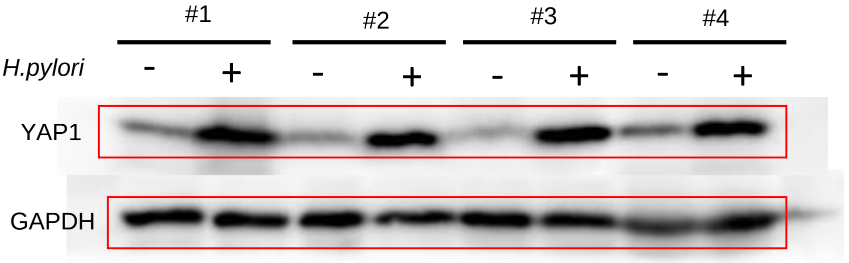

Figure 5L

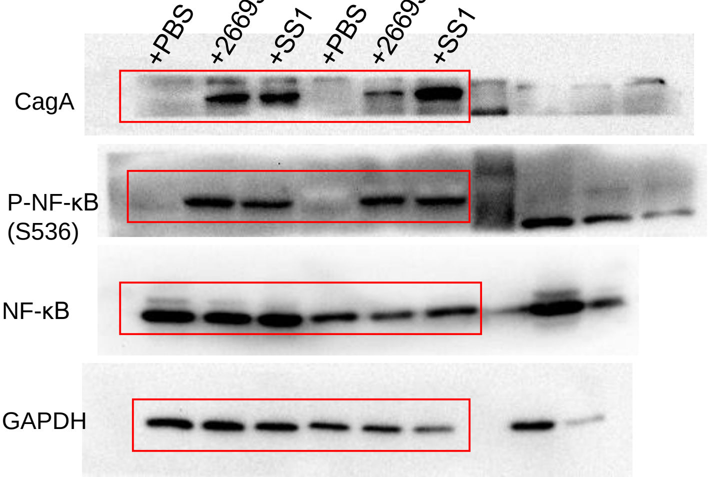

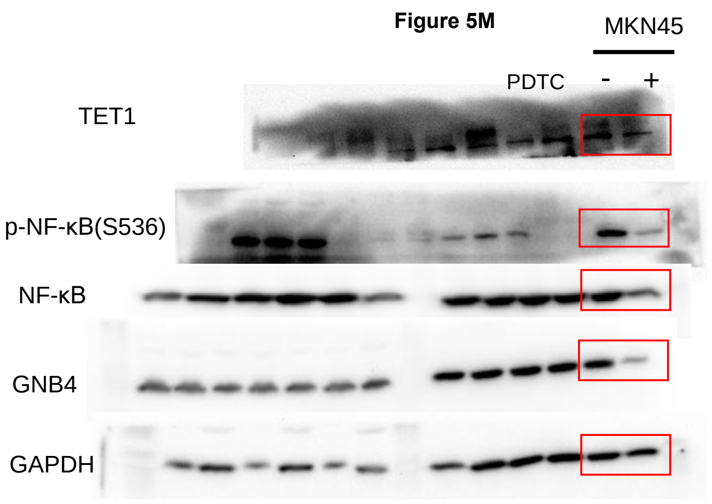
Figure 5M
MKN45
PDTC - +
TET1
p-NF-κB(S536)
NF-κB
GNB4
GAPDH

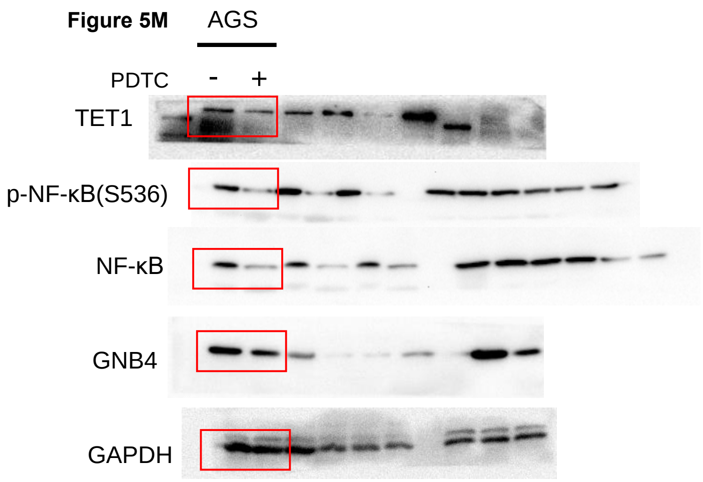
Figure 5M
AGS
PDTC - +
TET1
p-NF-κB(S536)
NF-κB
GNB4
GAPDH
